# Electronic Health Record Phenotyping of Pediatric Suicide-Related Emergency Department Visits

**DOI:** 10.1001/jamanetworkopen.2024.42091

**Published:** 2024-10-29

**Authors:** Juliet Beni Edgcomb, Loes Olde Loohuis, Chi-hong Tseng, Alexandra M. Klomhaus, Kristen R. Choi, Chrislie G. Ponce, Bonnie T. Zima

**Affiliations:** 1 Mental Health Informatics and Data Science Hub, Semel Institute for Neuroscience and Human Behavior, University of California Los Angeles; 2 Center for Neurobehavioral Genetics, Semel Institute for Neuroscience and Human Behavior, University of California, Los Angeles; 3 Department of Computational Medicine, David Geffen School of Medicine, University of California, Los Angeles; 4 Department of Human Genetics, David Geffen School of Medicine, University of California, Los Angeles; 5 Statistics Core, Department of Medicine, David Geffen School of Medicine at the University of California, Los Angeles; 6 University of California, Los Angeles School of Nursing; 7 Department of Health Policy and Management, Fielding School of Public Health, University of California, Los Angeles School of Nursing

## Abstract

**Importance:**

Suicide is a leading cause of death among young people. Accurate detection of self-injurious thoughts and behaviors (SITB) underpins equity in youth suicide prevention.

**Objectives:**

To compare methods of detecting SITB using structured electronic health information and measure algorithmic performance across demographics.

**Design, Setting, and Participants:**

This cross-sectional study used medical records among youths aged 6 to 17 years with at least 1 mental health–related emergency department (ED) visit in 2017 to 2019 to an academic health system in Southern California serving 787 000 unique individuals each year. Analyses were conducted between January and September 2023.

**Exposures:**

Multiexpert electronic health record review ascertained the presence of SITB using the Columbia Classification Algorithm of Suicide Assessment. Random forest classifiers with nested cross-validation were developed using (1) *International Statistical Classification of Diseases, Tenth Revision, Clinical Modification* (*ICD-10-CM*) codes for nonfatal suicide attempt and self-harm and chief concern and (2) all available structured data, including diagnoses, medications, and laboratory tests.

**Main Outcome and Measures:**

Detection performance was assessed overall and stratified by age group, sex, and race and ethnicity.

**Results:**

The sample comprised 2702 unique youths with an MH-related ED visit (1384 youths who identified as female [51.2%]; 131 Asian [4.8%], 266 Black [9.8%], 719 Hispanic [26.6%], 1319 White [48.8%], and 233 other race [8.6%]; median [IQR] age, 14 [12-16] years), including 898 children and 1804 adolescents. Approximately half of visits were related to SITB (1286 visits [47.6%]). Sensitivity of SITB detection using only codes and chief concern varied by age group and increased until age 15 years (6-9 years: 59.3% [95% CI, 48.5%-69.5%]; 10-12 years: 69.0% [95% CI, 63.8%-73.9%]; 13-15 years: 88.4% [95% CI, 85.1%-91.2%]; 16-17 years: 83.1% [95% CI, 79.1%-86.6%]), while specificity remained constant. The area under the receiver operating characteristic curve (AUROC) was lower among preadolescents (0.841 [95% CI, 0.815-0.867]) and male (0.869 [95% CI, 0.848-0.890]), Black (0.859 [95% CI, 0.813-0.905]), and Hispanic (0.861 [95% CI, 0.831-0.891]) youths compared with adolescents (0.925 [95% CI, 0.912-0.938]), female youths (0.923 [95% CI, 0.909-0.937]), and youths of other races and ethnicities (eg, White: 0.901 [95% CI, 0.884-0.918]). Augmented classification (ie, using all available structured data) outperformed classification with codes and chief concern alone (AUROC, 0.975 [95% CI, 0.968-0.980] vs 0.894 [95% CI, 0.882-0.905]; *P* < .001).

**Conclusions and Relevance:**

In this study, diagnostic codes and chief concern underestimated SITB prevalence, particularly among minoritized youths. These results suggest that priority on algorithmic fairness in suicide prevention strategies must extend to accurate detection of youths with suicide-related emergencies.

## Key Points

**Question:**

To what extent are self-injurious thoughts and behaviors (SITB) among youths reflected in structured electronic health record (EHR) information?

**Findings:**

In this cross-sectional study of 2702 youths with a mental health–related emergency department visit, multiexpert EHR review revealed that SITB-related diagnostic codes and chief concerns were not consistently assigned and were disproportionately omitted from visits of preadolescents, male youths, and Black and Hispanic youths with SITB. Detection was improved and discrepancies were reduced when classification algorithms leveraged additional structured information, such as psychiatric comorbidity, social vulnerability, and involuntary detainment.

**Meaning:**

This study found that youths with SITB did not consistently incur corresponding diagnostic codes or chief concerns and identified additional EHR characteristics associated with improved detection of these events.

## Teaser

This cross-sectional study compares the detection of self-injurious thoughts and behaviors among youths with emergency department visits by manual electronic health record vs random forest classifier review and by demographic group.

## Introduction

Suicide is a leading cause of death among young people.^1^ Children present for self-injurious thoughts and behaviors (SITB) with unprecedented frequency.^2^ Nationwide, 2.5% of high school students make a suicide attempt requiring medical treatment.^3^ More than 1 in 3 youths who died by suicide had a health care visit in the month before death, and nearly 9 in 10 (88%) had a visit within the prior year.^4^ Toward the goals of early detection and intervention, the National Strategy for Suicide Prevention calls for surveillance of suicide and self-harm events and use of data to inform action.^5^ The annual 8.2% increase in suicide deaths among preteens from 2008 to 2022^6^ underscores the need to ensure that surveillance methods accurately detect SITB events.

National surveillance efforts leverage standardized groupings of *International Statistical Classification of Diseases, Tenth Revision, Clinical Modification* (*ICD-10-CM*) codes and chief concern to detect nonfatal suicide attempts and intentional self-harm.^7^ There is uncertainty regarding whether codes are sufficiently sensitive and specific to detect SITB in adults^8^ or if additional data are needed to strengthen detection.^9,10,11^ For many pediatric conditions, detection improves when a broad range of electronic health record (EHR) data are jointly leveraged to find cases.^12,13,14^ In adults, natural language processing of clinical notes is associated with substantial improvement in sensitivity in screening suicidal behavior compared with diagnostic codes alone.^15^ Among adolescents, automated detection using clinical notes yields fair to good performance in finding suicidal behavior,^16,17,18^ but it is unclear how text-based approaches perform compared with structured data.^19,20^

Accurate surveillance of SITB in pediatric health care visits is complicated by 2 challenges. First, suicidal behavior varies strikingly across childhood,^21^ so a one-size-fits-all approach to detecting this behavior may not be valid. Cognition, judgment, impulsivity, and method availability vary with age,^22,23,24^ as do clinician schemas regarding the capabilities of preadolescents to comprehend the nature and finality of death.^25^ For example, a preteen who runs into traffic with stated intent to die could receive an injury, unspecified, or impulse control disorder diagnosis. Detection of SITB may clarify developmental trajectories of suicide, particularly given that nonsuicidal self-injury (NSSI) in preteens is a stronger predictor of future suicidal behavior than history of attempt.^26^ The second challenge lies in surmounting inequity in medical record data.^27,28^ Suicide, including among adolescents, is undercounted nonrandomly by sex, race and ethnicity, and method of death.^29,30^ Statistical models perform poorly in predicting suicide among Black and American Indian or Alaska Native individuals.^31^ In adults, intentional self-injury code accuracy varies by race and ethnicity, sex, and method.^32^ In children, 1 study described poorer detection of SITB-related emergencies among males and preteens, but small sample size precluded examination of racial disparities.^19^ Studies portend but have not directly examined differential misclassification of youths with SITB, with potential for higher misclassification risk among those who are members of racial and ethnic minority groups.

This study thus addresses the following 3 questions: To what extent are *ICD-10-CM* codes and chief concern accurate screening tools for identifying emergency department (ED) visits associated with SITB among children and adolescents? Is classifying visits using additional structured data associated with improved performance? Does misclassification vary by youth age, sex, and race and ethnicity? To answer these questions, we conducted a multiexpert clinical EHR review of 2702 ED visits by children and adolescents. We developed a series of random forest^33^ classifiers trained on structured data and manual review and then compared detection performance of each classifier by youth characteristics.

## Methods

### Design, Setting, and Study Population

This retrospective cross-sectional study was conducted at a large academic health care system in Southern California serving 787 000 unique individuals each year and used EHR data from 4 hospitals. The health system provides integrated care across more than 3.6 million annual outpatient visits and 79 500 emergency department visits per year. We included a random subset of individuals aged 6 to 17 years with at least 1 mental health (MH)–related ED visit between October 2017 and 2019 (eFigure 1 in Supplement 1). We defined MH-related ED visits as those associated with (1) a pediatric MH disorder, as specified by the Child and Adolescent Mental Health Disorders Classification System (CAMHD-CS),^34^ (2) an MH-related chief concern, (3) involuntary psychiatric detainment, or (4) a positive response to the nursing triage question “Does this patient have a primary psychiatric concern or suspicion of psychiatric illness?” We restricted the sample to the most recent visit for each youth to maximize the number of unique individuals. The study followed the Strengthening the Reporting of Observational Studies in Epidemiology (STROBE) reporting guideline. Analyses were conducted between January and September 2023. The University of California, Los Angeles Institutional Review Board approved this study and waived the requirement for informed consent under 45 CFR 46.116.

### Youth Characteristics

We use the term *children* to mean individuals aged 6 to 12 years and *adolescents* to mean individuals aged 13 to 17 years. Race and ethnicity and sex were determined from patient- or caregiver report as recorded in EHRs. Racial and ethnic categories were ascertained in alignment with Office of Management and Budget revisions to the Standards for the Classification of Federal Data on Race and Ethnicity: American Indian or Alaska Native, Asian, Black or African American, Hispanic or Latino (including Cuban, Mexican, Puerto Rican, South or Central American, or other Spanish culture or origin, regardless of race), Native Hawaiian or Other Pacific Islander, White, other, and multiple races. Due to low sample size, the category *other race and ethnicity* was used during stratification of model performance by demographic group and included American Indian or Alaska Native, Native Hawaiian or Pacific Islander, and individuals identifying with multiple races and ethnicities. Youth address was used to identify census tract of residence. Because neighborhood socioeconomic factors can influence quality of child MH care, we obtained Social Vulnerability Index scores^35^ and Area Deprivation Index state (decile) and national (percentile) rankings for each youth’s census tract^36^ (eMethods in Supplement 1).

### Encounter Characteristics

We focused on medical records from the ED visit. We defined the diagnostic code for SITB as the presence in any diagnostic field of 1 or more *ICD-10-CM* codes from the Centers for Disease Control and Prevention case surveillance definition of nonfatal suicide attempt and intentional self-harm.^37^ Diagnostic codes were grouped in 23 categories using the CAMHD-CS schema. A separate variable encoded presence of psychiatric comorbidity (defined as ≥1 CAMHD-CS category). We categorized chief concerns collected during triage as MH related and SITB related (eTable 1 in Supplement 1). We included psychotropic medications, laboratory tests related to overdose and urine drug screens, discharge disposition, and number of prior acute care visits in the past 90, 180, and 365 days. Encoding and aggregation are described in the eMethods in Supplement 1.

### Outcome Ascertainment

Deidentified notes from each visit were reviewed by 2 trained abstractors (including C.G.P.). Notes comprised physician, consultant, social worker, nurse, and MH detainment documentation. Abstractors labeled each visit for the presence and type of SITB using a modified version of the Columbia Classification Algorithm of Suicide Assessment (eMethods in Supplement 1).^38^ When abstractors disagreed, 2 board-certified child psychiatrists (J.B.E. and B.T.Z.) and a psychiatric nurse practitioner (K.R.C.) separately reviewed records to assign a classification. When clinicians disagreed, consensus discussion determined final case classifications.

### Statistical Analysis

To ascertain performance of existing case surveillance metrics by age group, bivariate contingency matrices were constructed comparing classification with suicide-related *ICD-10-CM* code or chief concern (either present) with manual EHR review. Random forest classifiers were developed using nested 10-fold cross-validation (eMethods in Supplement 1). Each fold trained a classifier with EHR data features and the outcome. The classifier assigned a probability of SITB, and the model classification was compared with manual EHR review classification to yield fit metrics. These were reported only on test data. Cross-validation variability was used to construct asymptotically exact CIs for the classifier’s test error.^39^ Estimated class was compared with manual review for all youths in each test fold and stratified by demographics. Classifiers were compared using DeLong tests.^40^

We trained classifiers on data from individuals of all ages, then applied them to estimate a single class (any SITB vs none) and multiclass classification using 1-vs-rest comparisons (attempts or preparatory acts, suicidal ideation, no suicidality, and NSSI). For the latter estimate, given the anticipated rarity of outcome, attempts and preparatory acts were grouped. Then, we explored training classifiers exclusively on children and exclusively on adolescents.

We trained and tested classifiers using 2 sets of features. No generative artificial intelligence or prompts were used. Performance was stratified by demographics. Model performance was compared with criterion-standard human review. The first set (case surveillance [CS]*)* comprised features reflecting existing surveillance definitions of nonfatal suicide-related outcomes and self-harm (ie, SITB-related diagnostic code and chief concern) and demographics. The second set (augmented case surveillance [aCS]*)* comprised these features plus youth and encounter characteristics described previously.

We imputed missing data through corresponding medians. Significance was set at 2-sided *P* < .05. Analyses were conducted in R statistical software version 4.3.2 (R Project for Statistical Computing) with roc.test and Python programming language version 3.12 (Python Software Foundation) with scikit-learn version 1.2.2. Classification models were developed using the same R and Python versions between January and September 2023. Code is available from the authorship team upon request.

## Results

### Sample Characteristics

We identified 2702 unique youths with an MH-related ED visit (1384 youths who identified as female [51.2%]; 131 Asian [4.8%], 266 Black [9.8%], 719 Hispanic [26.6%], 1319 White [48.8%], and 233 other race [8.6%]; median [IQR] age, 14 [12-16] years), including 898 children and 1804 adolescents. Sex varied by age group; 359 children were female (40.0%), and 1025 adolescents were female (56.8%). Table 1 presents sample characteristics by age group; additional characteristics are in eTable 2 in Supplement 1. Approximately half of visits were related to SITB (1286 visits [47.6%]). Raters agreed on SITB classification (2602 of 2702 classifications [96.3% agreement]; Cohen κ = 0.812). Among children, the most common MH-related diagnoses were attention-deficit/hyperactivity disorder (ADHD; 272 children [30.3%]), suicide or self-injury (266 children [29.6%]), anxiety disorders (207 children [23.1%]), depressive disorders (196 children [21.8%]), and other MH symptoms (193 children [21.5%]). By contrast, the most common MH-related diagnoses among adolescents were depressive disorders (747 adolescents [41.4%]), suicide or self-injury (684 adolescents [37.9%]), anxiety disorders (561 adolescents [31.1%]), ADHD (306 adolescents [17.0%]), and substance-related disorders (229 adolescents [12.7%]). Adolescents presented with suicide attempts more frequently than children (167 adolescents [9.3%] vs 58 children [6.5%]; χ^2^ = 6.15, *P* = .01) and more often reported suicide-related chief concerns (542 adolescents [30.0%] vs 1165 children [8.4%]; χ^2^ = 42.2; *P* < .001).

**Table 1.** Sample Characteristics by Age Group

Characteristic	ED visits, No. (%)		
	Total (N = 2702)	Ages 6-12 y (n = 898)	Ages 13-17 y (n = 1804)
Manual EHR review			
Any SITB	1286 (47.6)	427 (47.6)	859 (47.6)
Suicide attempt	225 (8.3)	58 (6.5)	167 (9.3)
Preparatory acts	189 (7)	71 (7.9)	118 (6.5)
Suicidal ideation	749 (27.7)	233 (25.9)	516 (28.6)
NSSI	543 (20.1)	181 (20.2)	362 (20.1)
Other reason for visit	1397 (51.7)	463 (51.6)	934 (51.8)
Not enough information	19 (0.7)	8 (0.9)	11 (0.6)
Age, median (IQR), y	14 (12-16)	11 (10-12)	16 (14-17)
Sex			
Female	1384 (51.2)	359 (40.0)	1025 (56.8)
Male	1318 (48.8)	539 (60.0)	779 (43.2)
Race and ethnicity			
Not Hispanic or Latino			
American Indian or Alaska Native	6 (0.2)	0	6 (0.3)
Asian	131 (4.8)	38 (4.2)	93 (5.2)
Black or African American	266 (9.8)	107 (11.9)	159 (8.8)
Native Hawaiian or Other Pacific Islander	2 (0.1)	0	2 (0.1)
White	1319 (48.8)	403 (44.9)	916 (50.8)
Other^a^	157 (5.8)	62 (6.9)	95 (5.2)
Multiple races	68 (2.5)	24 (2.7)	44 (2.4)
Hispanic or Latino	719 (26.6)	253 (28.2)	466 (25.8)
Unknown	34 (1.2)	11 (1.2)	23 (1.3)
Site			
Academic medical center	2021 (74.8)	728 (81.1)	1293 (71.7)
Community hospital	681 (25.2)	170 (18.9)	511 (28.3)
Disposition			
Discharged without hospitalization	1558 (57.7)	517 (57.6)	1041 (57.7)
General medical hospitalization	334 (12.4)	121 (13.5)	213 (11.8)
Psychiatric hospitalization	746 (27.6)	234 (26.1)	512 (28.4)
Chief concern			
Psychiatric (including suicide related)	1516 (56.1)	534 (59.5)	982 (54.4)
Suicide related	707 (26.2)	165 (18.4)	542 (30.0)
Legal status			
Involuntary mental health detainment	636 (23.5)	206 (22.9)	430 (23.8)
Voluntary			
ED diagnostic code category (CAMHD-CS)			
ADHD	578 (21.4)	272 (30.3)	306 (17.0)
Anxiety disorder	768 (28.4)	207 (23.1)	561 (31.1)
Autism spectrum disorder	321 (11.9)	155 (17.3)	166 (9.2)
Bipolar or related disorder	106 (3.9)	38 (4.2)	68 (3.8)
Communication disorder	25 (0.9)	18 (2)	7 (0.4)
Depressive disorder	943 (34.9)	196 (21.8)	747 (41.4)
Developmental delay or unspecified neurodevelopmental disorder	73 (2.7)	44 (4.9)	29 (1.6)
Disruptive, impulse control or conduct disorder	202 (7.5)	134 (14.9)	68 (3.8)
Feeding or eating disorder	60 (2.2)	9 (1)	51 (2.8)
Intellectual disability	48 (1.8)	20 (2.2)	28 (1.6)
Mental health symptom	369 (13.7)	193 (21.5)	176 (9.8)
Miscellaneous	141 (5.2)	59 (6.6)	82 (4.5)
Motor disorder	30 (1.1)	20 (2.2)	10 (0.6)
Neurocognitive disorder	38 (1.4)	12 (1.3)	26 (1.4)
Obsessive-compulsive or related disorder	121 (4.5)	39 (4.3)	82 (4.5)
Personality disorder	19 (0.7)	4 (0.4)	15 (0.8)
Schizophrenia spectrum or other psychotic disorder	90 (3.3)	28 (3.1)	62 (3.4)
Sexuality or gender identity disorder	24 (0.9)	2 (0.2)	22 (1.2)
Specific learning disorder	18 (0.7)	9 (1)	9 (0.5)
Substance related or addictive disorder	237 (8.8)	8 (0.9)	229 (12.7)
Suicide or self-injury	950 (35.2)	266 (29.6)	684 (37.9)
Trauma or stressor-related disorder	167 (6.2)	65 (7.2)	102 (5.7)
Acute care use			
Prior ED use (≥1 visit)			
Past 90 d	601 (22.2)	194 (21.6)	407 (22.6)
Past 180 d	967 (35.8)	309 (34.4)	658 (36.5)
Past 365 d	1577 (58.4)	457 (50.9)	1120 (62.1)
Prior medical hospitalization (≥1 visit)			
Past 90 d	97 (3.6)	31 (3.5)	66 (3.7)
Past 180 d	166 (6.1)	50 (5.6)	116 (6.4)
Past 365 d	279 (10.3)	80 (8.9)	199 (11)
Prior psychiatric hospitalization (≥1 visit)			
Past 90 d	99 (3.7)	36 (4)	63 (3.5)
Past 180 d	173 (6.4)	58 (6.5)	115 (6.4)
Past 365 d	260 (9.6)	79 (8.8)	181 (10)
Medication received during ED visit			
Antidepressant	549 (20.3)	143 (15.9)	406 (22.5)
Antiepileptic	117 (4.3)	46 (5.1)	71 (3.9)
Antihistamine	100 (3.7)	29 (3.2)	71 (3.9)
Antipsychotic	439 (16.2)	159 (17.7)	280 (15.5)
Anxiolytic	239 (8.8)	47 (5.2)	192 (10.6)
Hypnotic or sedative	43 (1.6)	16 (1.8)	27 (1.5)
Lithium	42 (1.6)	11 (1.2)	31 (1.7)
Psychostimulant	233 (8.6)	126 (14)	107 (5.9)
Injectable medication	84 (3.1)	14 (1.6)	70 (3.9)
Social Vulnerability Index total, median (IQR)	0.37 (0.18-0.64)	0.42 (0.19-0.71)	0.366 (0.18-0.62)
Socioeconomic status, median (IQR)^b^	0.29 (0.13-0.56)	0.30 (0.14-0.66)	0.29 (0.22-0.67)
Household composition and disability, median (IQR)	0.23 (0.11-0.42)	0.27 (0.12-0.47)	0.221 (0.09-0.42)
Minority status and language, median (IQR)	0.684 (0.55-0.84)	0.72 (0.55-0.86)	0.67 (0.54-0.83)
Housing type and transportation, median (IQR)	0.507 (0.24-0.75)	0.50 (0.24-0.77)	0.51 (0.25-0.75)
Area Deprivation Index			
State ranking, median (IQR)	2 (1-5)	3 (1-5)	2 (1-4)
National ranking, median (IQR)	5 (2-12)	6 (2-14)	4 (2-11)

Abbreviations: ADHD, attention-deficit/hyperactivity disorder; CAMHD-CS, Child and Adolescent Mental Health Disorders Classification System; ED, emergency department; EHR, electronic health record; NSSI, nonsuicidal self-injury; SITB, self-injurious thoughts and behaviors.

^a^ There was a separate race and ethnicity category item for other.

^b^ Separate variables encoded the overall Social Vulnerability Index score and subdomains: socioeconomic status, household composition and disability, minority status and language, and housing type and transportation. Each is scored on a scale of 0 to 1, with higher scores indicating greater vulnerability.

### Classification of Self-Injurious Thoughts and Behaviors

The accuracy of *ICD-10-CM* codes and chief concern varied by age (Table 2). The youngest children were most poorly detected, and accuracy increased with age (6-9 years: 81.2% [95% CI, 75.3%-86.3%]; 10-12 years: 84.6% [95% CI, 81.7%-87.2%]; 13-17 years: 92.4% [95% CI, 90.5%-94.0%]). Sensitivity increased until age 15 years (6-9 years: 59.3% [95% CI, 48.5%-69.5%]; 10-12 years: 69.0% [95% CI, 63.8%-73.9%]; 13-15 years: 88.4% [95% CI, 85.1%-91.2%]; 16-17 years: 83.1% [95% CI, 79.1%-86.6%]), while specificity remained similar across age groups.

**Table 2.** Detection of Self-Injurious Thoughts and Behaviors vs Manual Electronic Health Record Review^a^

Classification outcome	Visits, No. (%) (N = 2702)			
	Aged 6-9 y (n = 208)	Aged 10-12 y (n = 690)	Aged 13-15 y (n = 861)	Aged 16-17 y (n = 943)
True positive	54 (26.0)	232 (33.6)	404 (46.9)	334 (35.4)
False positive	2 (1.0)	2 (0.3)	12 (1.4)	4 (0.4)
False negative	37 (17.8)	104 (15.1)	53 (6.2)	68 (7.2)
True negative	115 (55.3)	352 (51.0)	392 (45.5)	537 (56.9)
Metric (95% CI), %				
Sensitivity	59.3 (48.5-69.5)	69.0 (63.8-73.9)	88.4 (85.1-91.2)	83.1 (79.1-86.6)
Specificity	98.4 (94.0-99.8)	99.4 (98.0-99.3)	97.0 (94.9-98.5)	99.3 (98.1-99.8)
Accuracy	81.2 (75.3-86.3)	84.6 (81.7-87.2)	92.4 (90.5-94.1)	92.4 (90.5-94.0)

^a^ The contingency matrix compares the number and percentage of youths with self-injurious thoughts and behaviors vs none using suicide or self-harm related diagnostic code or chief concern (either present) vs manual electronic health record review using the Columbia Classification Algorithm for Suicide Assessment.

**Figure 1.**
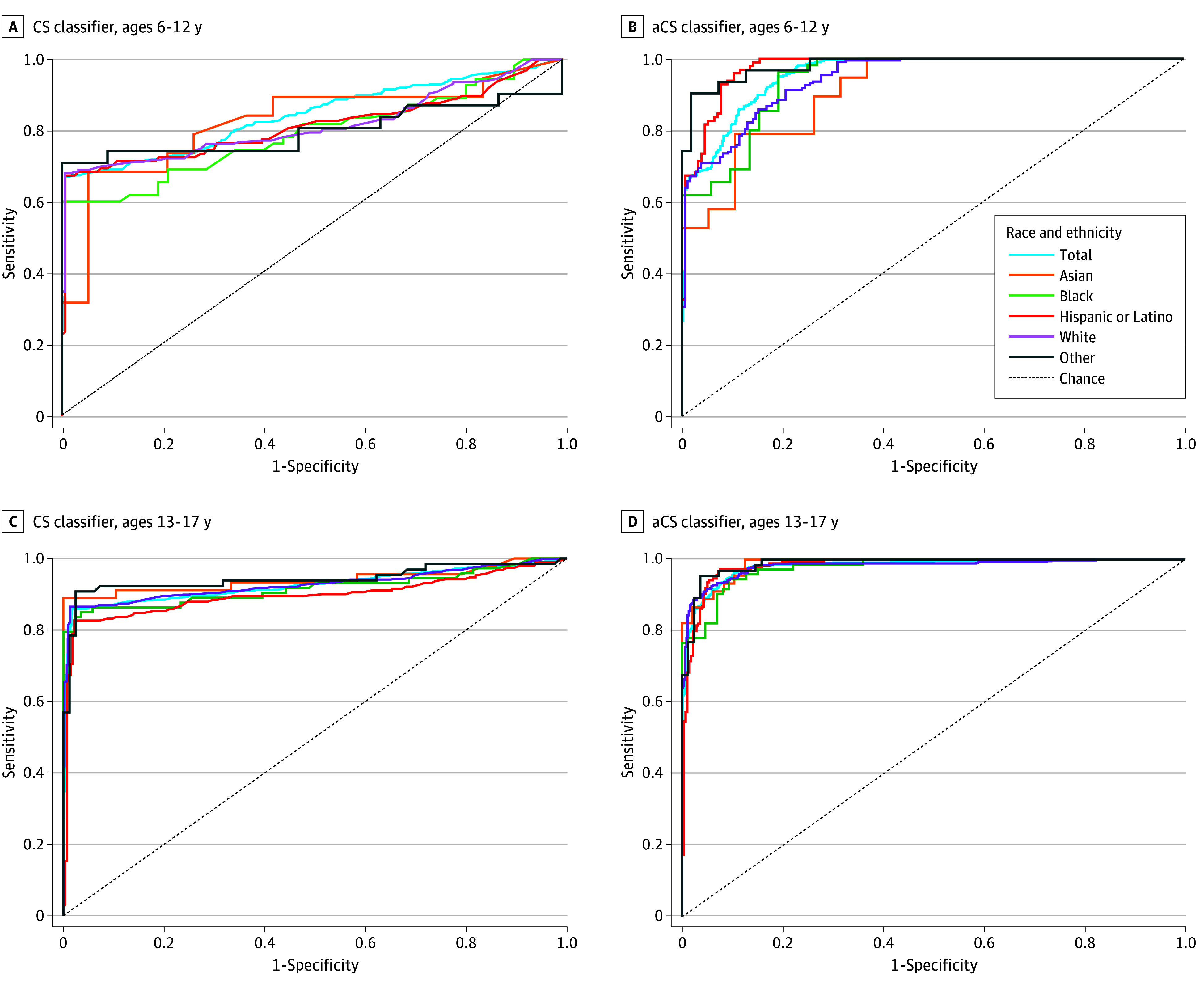
Area Under the Receiver Operating Characteristic Curves (AUROCs) The diagram is a composite of AUROC curves for case surveillance (CS) and augmented case surveillance (aCS) random forest classifiers for detection of self-injurious thoughts and behaviors among youths aged 6 to 12 years (A-B) and 13 to 17 years (C-D). Features of CS classifiers include the Centers for Disease Control and Prevention Case Surveillance *International Statistical Classification of Diseases, Tenth Revision, Clinical Modification *(*ICD-10-CM*) code list for nonfatal suicide attempt and self-harm, suicide-related chief concern, and individual age, sex, and race and ethnicity. Features of aCS classifiers add structured data elements from individual electronic health records, including medications administered, laboratory testing, emergency department disposition, and mental health detainment. For each age group, classifier performance is stratified by race and ethnicity.

**Figure 2.**
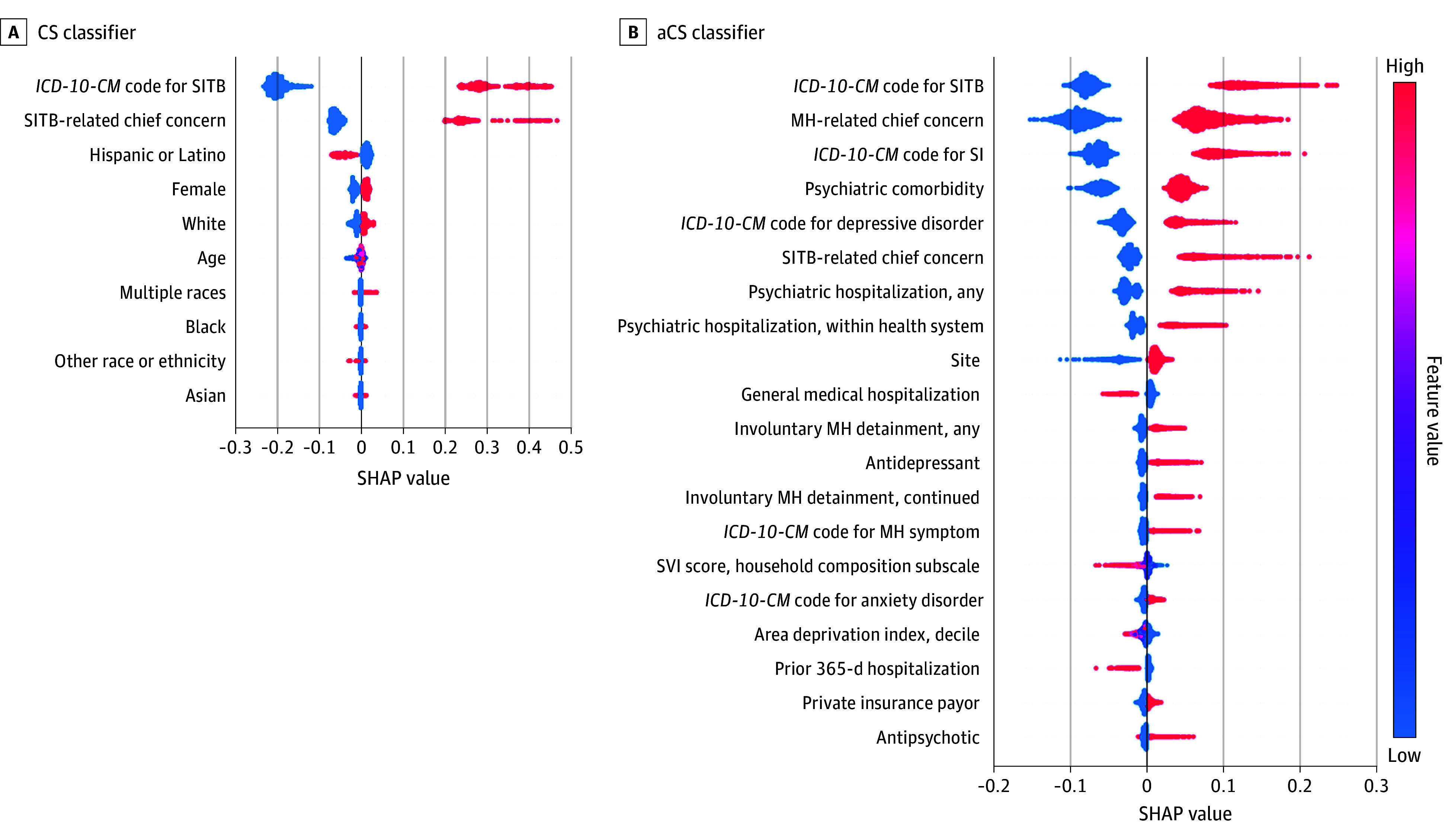
Shapley Additive Explanation (SHAP) Plots The diagram is a composite of SHAP plots for case surveillance (CS) and augmented case surveillance (aCS) random forest classifiers for detection of self-injurious thoughts and behaviors (SITB) among youths aged 6 to 17 years. Features of CS classifiers include the Centers for Disease Control and Prevention Case Surveillance *International Statistical Classification of Diseases, Tenth Revision, Clinical Modification *(*ICD-10-CM*) code list for nonfatal suicide attempt and self-harm, suicide-related chief concern, and individual age, sex, and race and ethnicity. Features of aCS classifiers add structured data elements from individual electronic health records, including medications administered, laboratory testing, emergency department disposition, and mental health (MH) detainment. For each classifier, features are shown in descending order of feature importance. SI indicates suicidal ideation; SVI, Social Vulnerability Index.

The aCS classifier outperformed the CS classifier, with greater AUROC (0.975 [95% CI, 0.968-0.980] vs 0.894 [95% CI, 0.882-0.905]; *P* < .001) (Table 3; Figure 1). At a 50% probability cutoff value, the aCS classifier was more sensitive (92.0% [95% CI, 91.5-93.5%] vs 79.6% [95% CI, 77.1%-82.1%]) and less specific (89.4% [95% CI, 87.7%-91.1%] vs 98.5% [95% CI, 97.9%-99.2%) than the CS classifier. Accuracy, sensitivity, and specificity metrics and fit metrics demonstrating comparable performance of the random forest to lasso logistic regression are presented in eTable 3 in Supplement 1. Figure 2 depicts Shapley additive explanation plots with features of greatest classification importance. Features varied by classifier (CS classifier: SITB-related diagnostic code and SITB-related chief concern, followed by youth demographics; aCS classifier: SITB-related diagnostic code, MH-related chief concern, suicidal ideation diagnostic code [R45.851], psychiatric comorbidity, and depressive disorder diagnostic code).

**Table 3.** Classifier Performance

Characteristic	AUROC (95% CI)								
	Total (N = 2702)			Aged 6-12 y (n = 898)			Aged 13-17 y (n = 1804)		
	CS	aCS	*P* value^a^	CS	aCS	*P* value	CS	aCS	*P* value
All	0.894 (0.882-0.905)	0.975 (0.968-0.980)	<.001	0.841 (0.815-0.867)	0.956 (0.942-0.97)	<.001	0.925 (0.912-0.938)	0.981 (0.974-0.988)	<.001
Sex									
Female	0.923 (0.909-0.937)	0.985 (0.979-0.991)	<.001	0.88 (0.845-0.915)	0.979 (0.964-0.994)	<.001	0.939 (0.924-0.954)	0.987 (0.98-0.994)	<.001
Male	0.869 (0.848-0.89)	0.961 (0.949-0.973)	<.001	0.814 (0.776-0.852)	0.939 (0.917-0.961)	<.001	0.904 (0.88-0.928)	0.972 (0.959-0.985)	<.001
Race and ethnicity									
Asian	0.895 (0.838-0.952)	0.970 (0.940-1.000)	.005	0.824 (0.689-0.959)	0.911 (0.813-1.009)	.15	0.940 (0.889-0.991)	0.987 (0.963-1.011)	.08
Black	0.859 (0.813-0.905)	0.965 (0.942-0.988)	<.001	0.791 (0.706-0.876)	0.941 (0.895-0.987)	<.001	0.917 (0.870-0.964)	0.977 (0.952-1.002)	.008
Hispanic or Latino	0.861 (0.831-0.891)	0.980 (0.968-0.992)	<.001	0.812 (0.755-0.869)	0.975 (0.953-0.997)	<.001	0.896 (0.864-0.928)	0.982 (0.969-0.995)	<.001
White	0.901 (0.884-0.918)	0.973 (0.964-0.982)	<.001	0.815 (0.774-0.856)	0.943 (0.920-0.966)	<.001	0.927 (0.91-0.944)	0.981 (0.972-0.99)	<.001
Other^b^	0.895 (0.850-0.940)	0.986 (0.969-1.003)	.003	0.799 (0.695-0.903)	0.982 (0.948-1.016)	<.001	0.942 (0.900-0.984)	0.988 (0.969-1.007)	.03

Abbreviations: aCS, augmented case surveillance classifier; AUROC, area under the receiver operating characteristic curve; CS, case surveillance classifier.

^a^ The DeLong test *P* value is given for the difference in AUROC between CS and aCS classifiers.

^b^ Includes American Indian or Alaska Native, Native Hawaiian or Other Pacific Islander, and multiple races and ethnicities, excluding unknown.

The performance of CS and aCS classifiers, as measured by AUROC, was higher in adolescents (CS: 0.925 [95% CI, 0.912-0.938]; aCS: 0.981 [95% CI, 0.974-0.988]) compared with children (CS: 0.841 [95% CI, 0.815-0.867]; aCS: 0.956 [95% CI, 0.942-0.970]). Detection across adolescents and children was improved with aCS classification (*P* < .001 for both) (Table 3).

As measured by AUROC when stratified by sex, male youths (CS: 0.869 [95% CI, 0.848-0.890]; aCS: 0.961 [95% CI, 0.949-0.973]) were more poorly detected than female youths (CS: 0.923 [95% CI, 0.909-0.937]; aCS: 0.985 [95% CI, 0.979-0.991]) (eFigure 2 in Supplement 1). As measured by AUROC, both classifiers detected male children (CS: 0.814 [95% CI, 0.776-0.852]; aCS: 0.939 [95% CI, 0.917-0.961]) more poorly than male adolescents (CS: 0.904 [95% CI, 0.880-0.928]; aCS: 0.972 [95% CI, 0.959-0.985]). Male children were more poorly detected compared with female children as measured by AUROC with both CS (0.814 [95%CI 0.776-0.852] vs 0.880 [95% CI, 0.845-0.915]) and aCS (0.939 [95%CI 0.917-0.961 vs 0.979 [95% CI, 0.964-0.994]) classifiers. Detection was highest among female adolescents as measured by AUROC (CS: 0.939 [95% CI, 0.924-0.954]; aCS: 0.987 [95% CI, 0.980-0.994]). For both sexes and age groups, the aCS classifier outperformed the CS classifier (*P* < .001) (Table 3).

Detection varied by race and ethnicity, with the most errors in detection among Black and Hispanic youths as measured by AUROC overall (eg, CS: 0.859 [95% CI, 0.813-0.905] among Black, 0.861 [95% CI, 0.831-0.891] among Hispanic, and 0.901 [95% CI, 0.884-0.918] among White youths) (Table 3). Augmented classification was associated with reduced variation in performance by race and ethnicity, although variation remained. Across groups, classifiers detected children more poorly as shown by AUROC (CS range, 0.791-0.824; aCS range, 0.911-0.982) than adolescents (CS range, 0.896-0.942; aCS range, 0.977-0.988) (Table 3; Figure 1). When stratified by age group and race and ethnicity, the aCS classifier had a greater AUROC compared with the CS classifier among adolescents (eg, Black: 0.977 [95% CI, 0.952-1.002] vs 0.917 [95% CI, 0.870-0.964]; *P* = .008; Hispanic or Latino: 0.982 [95% CI, 0.969-0.995] vs 0.896 [95% CI, 0.864-0.928]; *P* < .001; White: 0.981 [95% CI, 0.972-0.990] vs 0.927 [95% CI, 0.910-0.944]; *P* < .001) and children (eg, Black: 0.941 [95% CI, 0.895-0.987] vs 0.791 [95% CI, 0.706-0.876]; *P* < .001; Hispanic or Latino: 0.975 [95% CI, 0.953-0.997] vs 0.812 [95% CI 0.755-0.869]; *P* < .001; White: 0.943 [95% CI, 0.920-0.966] vs. 0.815 [95% CI, 0.744-0.856]; *P* < .001) (Table 3). CS and aCS classifier performance did not differ significantly among Asian youth.

Performance did not significantly differ when classifiers were trained separately by age group and then applied to that age group vs when trained on data from all individuals. Results are described in as metrics in eTable 4, AUROC curves in eFigure 3, and Shapley additive explanation plots in eFigure 4 in Supplement 1.

### Multiclass Classification of Ordinal Suicidality and NSSI

Augmented classification was associated with increased detection of ideation, preparatory acts and attempts, and NSSIs (eTable 5 in Supplement 1). AUROC curves by classifier and type are presented in eFigure 5 in Supplement 1. As shown by AUROCs, the aCS classifier compared with the CS classifier had improved detection of attempts and acts (0.859 [95% CI, 0.846-0.873] vs 0.792 [95% CI, 0.776-0.807]; *P* < .001), ideation (0.868 [95% CI, 0.855-0.881] vs 0.733 [95% CI, 0.716-0.750]; *P* < .001), and NSSIs (0.801 [95% CI, 0.786-0.816] vs 0.638 [95% CI, 0.712-0.745]; *P* < .001). Both classifiers yielded lower detection performance among children compared with adolescents. When stratified by age group, the aCS classifier outperformed the CS classifier (eTable 5 in Supplement 1).

## Discussion

In this cross-sectional study, multi-expert clinical EHR review revealed that children and adolescents with SITB did not consistently receive a corresponding diagnostic code or chief concern. Children, male youths, and Black and Hispanic youths with SITB were less likely to receive suicide-related codes or chief concern compared with adolescents, female youths, and youths of other races and ethnicities. Detection improved and variation attenuated when we used a classifier that considered additional structured data from the visit. Increased sensitivity contributed to the improved performance of the augmented classifier, and the features associated with improved sensitivity included psychiatric comorbidity, depression diagnostic codes, hospital visits, neighborhood vulnerability score, and involuntary detainment.

While diagnostic code and chief concern detected SITB in teens, these indicators had relatively low sensitivity among preteens (69.0%), and among children aged 6 to 9 years, sensitivity was poor (59.3%). Relying on measures that substantially underestimate SITB may obfuscate epidemiologic trends and individual risk estimation in childhood. This detection gap is particularly concerning given that 1 in 6 preadolescent children with suicidal ideation transitions to attempting suicide^25^ and that prevention of trajectories toward escalation in childhood is a prevention target area.^22^ Augmented classification was associated with substantial attenuation in the age gap in detection, suggesting that structured data may help clarify preadolescent trajectories of suicidality.

Classifiers less accurately detected male compared with female youths. Age and sex interacted such that the AUROC was lowest among male children and highest among female adolescents. Augmented classifiers had improved performance and reduced variation across all age-sex groups. Male children die by suicide and engage in NSSIs more frequently than female children, while sex is uncorrelated with ideation and attempts in preadolescence.^25^ The predominance of detection errors among male children belies their risk of omission from predictive models of suicidal behavior and epidemiologic surveillance.

Systematic errors in surveillance extended to race and ethnicity. These findings echo a growing literature calling evaluation for algorithmic fairness, that is, measures to protect against bias, discrimination, or inequity.^41,42^ Among youths with evidence of SITB on manual review, clinicians less frequently assigned corresponding codes and chief concerns to Black and Hispanic youths compared with youths of other races and ethnicities. Classifiers built exclusively on these metrics underdetected SITB among Black and Hispanic youths, especially children. Augmented classification had attenuated detection racial disparities, but disparities remained. The recent increase in Black youth suicide makes these errors in classification particularly concerning.^43,44^

Phenotypes impact policymaking around potential treatments, allocation of resources, and, consequently, the health of populations. Our findings support the need to establish best practices for evaluating phenotype definitions of SITB in youths and to ground these practices in algorithmic fairness metrics.^45^ Several factors, such as underlying psychopathology, reimbursement practices, stigma, and clinician biases, may explain why youths do not receive suicide-related codes and chief concerns. For example, Black preadolescents are less likely to report suicidal thoughts compared with other preadolescents in other racial groups, despite equivalent lifetime histories of past attempts.^46^ Another example is the potential moderating effect of externalizing behaviors, such as impulsivity and aggression, which are more common among male children^47^ and may diminish the perceived salience of suicidality.^47^

Existing case surveillance definitions combine nonfatal suicide attempts and intentional self-harm.^37^ However, research fidelity and clinical risk stratification require distinguishing these entitites.^48^ In this study, codes and chief concern best detected attempts and preparatory acts, while underestimating ideation and NSSIs. These findings reflect previous observations that severe illness phenotypes receive codes more often than subsyndromic or mild presentations.^49,50^ They also suggest an opportunity to strengthen detection of early signs of suicidality to support understanding of trajectories of risk prior to attempt.^32^

### Limitations

This study has several limitations. First, we cannot exclude the selection bias associated with youths who received care in the studied health system. Second, chief concern was labeled by nursing triage without free-text self-report. While there is a strong potential for text-based analysis to accelerate detection, we intentionally chose to restrict indicators to structured data. Structured data are commonly used as inclusion criteria to select populations for whom notes will be extracted; children not detected by structured data risk being entirely omitted. Manual EHR review of thousands of records may be infeasible to replicate for other populations. Our findings suggest that screening records with focused structured data beyond suicide-related diagnostic codes and concerns may be associated with improved sensitivity. Third, we restricted analyses to the most recent visit by each youth, and thus conclusions are based on a sample that equally weights youths with repeat visits and those with single visits. Fourth, although we explored multiclass classification with preparatory acts and attempts as separate outcomes, neither CS nor aCS models discriminated these behaviors, perhaps because there are few indicators in structured data to differentiate when the potential for harm^38^ has begun. Variables relevant to suicide risk (eg, gender identity) were not examined owing to poor availability or missingness. Fifth, notes may imperfectly represent the reality of the clinical scenario. These are prone to biases of the writer^51^ and the extent to which the child or adolescent felt comfortable to disclose information.^52,53,54^

## Conclusions

In this cross-sectional study, youths with suicide-related emergencies did not consistently incur corresponding diagnostic codes or chief concerns. Relying solely on these indicators missed SITB events, especially among preadolescent children and male, Black, and Hispanic youths. Structured data alone distinguished some populations, such as adolescent females with suicidality. However, more nuanced indicators are necessary for other populations, such as male children with NSSIs. Shifts in clinician practices could increase sensitivity to detect suicide-related emergencies with diagnostic codes and chief concerns. In the interim, without accurate and equitable phenotype definitions, suicide prevention strategies will inadequately target the populations they aim to serve.

## Notes

Supplement 1.
eMethods.
**eTable 1.** Mental Health–Related Chief Concerns in Structured Data Fields **eTable 2.** Full Sample Characteristics by Age Group **eTable 3.** Classifier Performance With Table of Counts and Fit Metrics by Age Group, Sex, and Race and Ethnicity **eTable 4.** Delong Tests Comparing Augmented Case Surveillance Performance by Age Group of Training Set **eTable 5.** Comparison of Performance for Detection of Self-Injurious Thoughts and Behaviors With Table of Counts and Fit Metrics by Type and by Age Group **eFigure 1.** Flowchart for Study Inclusion **eFigure 2.** Area Under the Receiver Operating Characteristic Curves by Sex **eFigure 3.** Area Under the Receiver Operating Characteristic Curves by Age Group of Training Set **eFigure 4.** Shapley Additive Explanation Plots by Age Group of Training Set **eFigure 5.** Area Under the Receiver Operating Characteristic Curves for Detection of Self-injurious Thoughts and Behavior by Type

Supplement 2.
Data Sharing Statement
