# Supplementary material for: Electronic Health Record Phenotyping of Pediatric Suicide-Related Emergency Department Visits
Source: JAMA Netw Open. 2024 Oct 29;7(10):e2442091. doi: 10.1001/jamanetworkopen.2024.42091 (PMC11522940; doi:10.1001/jamanetworkopen.2024.42091)
Supplement: Supplement 1. — eMethods. eTable 1. Mental Health–Related Chief Concerns in Structured Data Fields eTable 2. Full Sample Characteristics by Age Group eTable 3. Classifier Performance With Table of Counts and Fit Metrics by Age Group, Sex, and Race and Ethnicity eTable 4. Delong Tests Comparing Augmented Case Surveillance Performance by Age Group of Training Set eTable 5. Comparison of Performance for Detection of Self-Injurious Thoughts and Behaviors With Table of Counts and Fit Metrics by Type and by Age Group eFigure 1. Flowchart for Study Inclusion eFigure 2. Area Under the Receiver Operating Characteristic Curves by Sex eFigure 3. Area Under the Receiver Operating Characteristic Curves by Age Group of Training Set eFigure 4. Shapley Additive Explanation Plots by Age Group of Training Set eFigure 5. Area Under the Receiver Operating Characteristic Curves for Detection of Self-injurious Thoughts and Behavior by Type [file jamanetwopen-e2442091-s001.pdf]

Supplemental Online Content

Edgcomb JB, Olde Loohuis L, Tseng C, et al. Electronic health record phenotyping of pediatric suicide-related emergency department visits. *JAMA Netw Open*. 2024;7(10):e2442091. doi:10.1001/jamanetworkopen.2024.42091

eMethods.

eTable 1. Mental Health–Related Chief Concerns in Structured Data Fields

eTable 2. Full Sample Characteristics by Age Group

eTable 3. Classifier Performance With Table of Counts and Fit Metrics by Age Group, Sex, and Race and Ethnicity

eTable 4. DeLong Tests Comparing Augmented Case Surveillance Performance by Age Group of Training Set

eTable 5. Comparison of Performance for Detection of Self-Injurious Thoughts and Behaviors With Table of Counts and Fit Metrics by Type and by Age Group

eFigure 1. Flowchart for Study Inclusion

eFigure 2. Area Under the Receiver Operating Characteristic Curves by Sex

eFigure 3. Area Under the Receiver Operating Characteristic Curves by Age Group of Training Set

eFigure 4. Shapley Additive Explanation Plots by Age Group of Training Set

eFigure 5. Area Under the Receiver Operating Characteristic Curves for Detection of Self-injurious Thoughts and Behavior by Type

This supplemental material has been provided by the authors to give readers additional information about their work.

© 2024 Edgcomb JB et al. *JAMA Network Open.*

eMethods

Chart Abstraction:

Consensus review of charts: When staff research abstractors selected differing primary or secondary classifications for presence or type of self-injurious thoughts or behaviors (SITB), these visits were flagged for further review. These flagged visits were blindly reviewed by the three clinicians (J.E., B.Z., K.C.) who each separately assigned a classification. When ≥2 clinicians agreed on a classification, this was used as the final classification. When all clinicians disagreed, the three clinicians and two staff research abstractors met for four one-hour deliberation meetings to present justification for their classification and areas of uncertainty. In all cases, this discussion yielded consensus determination of a final classification.

Outcome ascertainment: Manual chart review and coding for presence and type of SITB was conducted using an adapted form of the Columbia Classification Algorithm for Suicide Assessment (C-CASA). We adapted the C-CASA in the following ways: First, we recorded NSSI in a separate field to avoid obfuscating co-occurring NSSI and suicidality. Second, we combined 'Self-injurious behavior, suicidal intent unknown' and 'Self-injurious behavior, no suicidal intent' into a single category given previous reports (Posner, 2007) of poor inter-rater reliability in distinguishing these (Intraclass Correlation [ICC] 0.59-0.67). Using these adaptations to the C-CASA categories, we assigned each encounter one of the following primary classifications: (1) completed suicide, (2) suicide attempt, (3) preparatory acts toward imminent suicidal behavior, (4) suicidal ideation, (5) non-suicidal self-injury, (6) other, no deliberate self-harm, (7) not enough information. We also assigned each encounter one of the following secondary classifications: (1) non-suicidal self-injury present, or (2) non-suicidal self-injury absence.

Child and Encounter Characteristics:

Diagnoses: International Classification of Diseases Version 10 clinical modification (ICD-10-CM) codes in any diagnostic field and associated with the emergency department (ED) encounter were encoded into categories using the Child and Adolescent Mental Health Disorders Classification System (CAMHD-CS). Additional categories were presence of ICD-10-CM code for suicidal ideation (R45.851), presence of comorbidity (≥ 1 CAMHD-CS category), and presence of a medical diagnosis other than listed in CAMHD-CS. The CAMHD-CS category for suicide and self-harm aligns with the ICD-10-CM list as described in Hedegaard H, Schoenbaum M, Claassen C, Crosby A, Holland K, Proescholdbell S. Issues in developing a surveillance case definition for nonfatal suicide attempt and intentional self-harm using International Classification of Diseases, Tenth Revision, Clinical Modification (ICD-10-CM) coded data. National health statistics reports. 2018;(108):1-19.

Medications: Psychotropic medications administered during the visit were categorized using Anatomical Therapeutic Chemical (ATC) classes into 8 groups: antidepressants (amitriptyline, bupropion, citalopram, clomipramine, desvenlafaxine, doxepin, duloxetine, escitalopram, fluoxetine, fluvoxamine, imipramine, mirtazapine, paroxetine, sertraline, trazodone, venlafaxine, vilazodone, vortioxetine), antiepileptics (carbamazepine, divalproex, ethosuximide, lamotrigine, levetiracetam, oxcarbazepine, phenobarbital, phenytoin, valproate, valproic), antihistamines, antipsychotics (aripiprazole, asenapine, brexpiprazole, cariprazine, chlorpromazine, chlorpromazine, fluphenazine, haloperidol, lurasidone, olanzapine, paliperidone, prochlorperazine, promethazine, quetiapine, risperidone, ziprasidone), anxiolytics (alprazolam, buspirone, chlordiazepoxide, clobazam, clonazepam, diazepam, hydroxyzine, lorazepam, temazepam, triazolam), hypnotics and sedatives (melatonin, zaleplon, zolpidem, zonisamide), lithium, and psychostimulants (amphetamine, amphetamine-dextroamphetamine, dexmethylphenidate, dextroamphetamine, guanfacine, lisdexamfetamine, methylphenidate, modafinil), and injectable medications with high probability of psychiatric indication (chlorpromazine, diazepam, haloperidol, lorazepam, olanzapine, and prochlorperazine)

© 2024 Edgcomb JB et al. *JAMA Network Open.*

Laboratory tests: Laboratory tests related to overdose (serum acetaminophen > 10mcg/mL, salicylates > 3.0mg/dL, benzodiazepines > 0 ng/L, and tricyclics > 3.0ng/mL), urine drug screen results (positive, negative), serum alcohol (> 15mg/dL) were encoded to binary variables.

Disposition: Emergency department disposition was categorized as discharged, general medical hospitalization, psychiatric hospitalization (any, within, and outside of health system), transferred, eloped, left without being seen, deceased, and sent to labor and delivery.

Insurance status: Insurance status at time of emergency department visit was grouped as public, private, or none/unknown.

Prior care use: As most children receive outpatient mental health care in the community, we limited information on prior encounters to acute care encounters. We coded number of ED visits, general medical hospitalizations, and psychiatric hospitalizations as the number of each visit type occurring in the past 90-, 180-, and 365-days within the health system.

Legal status: Legal status was defined as presence of a 5585 mental health detainment order (any, none) and an additional binary variable coded whether the detainment was continued following mental health evaluation.

Area Deprivation Index: State decile and national percentile linked by census tract of child's home address.

Social Vulnerability Index: The Social Vulnerability Index (SVI) primarily utilizes data from the U.S. Census Bureau's American Community Survey (ACS) through a combination of various socioeconomic indicators linked by census tract to child's home address. The SVI uses 16 U.S. Census Bureau variables to determine the social vulnerability of each census tract. Separate variables encoded the overall SVI score and subdomains: socioeconomic status, household composition and disability, minority status and language, housing type and transportation. Each is scored on a scale of 0 to 1, with higher scores indicating greater vulnerability.

Site: Encoded as a binary variable representing primary location of ED visit: quaternary care academic medical center, community hospital.

Prior care use: Encoded as number of ED visits, general medical hospitalizations, and psychiatric hospitalizations in the past 90, 180, and 365 days.

Race and ethnicity: Patient or parent-reported racial and ethnic categories in alignment with OMB Revisions to the Standards for the Classification of Federal Data on Race and Ethnicity (NOT-OD-15-053): American Indian or Alaska Native, Asian, Black or African American, Hispanic or Latino (including Cuban, Mexican, Puerto Rican, South or Central American, or other Spanish culture or origin, regardless of race), Native Hawaiian or Other Pacific Islander, and White. Due to low sample size, during comparison of model performance across demographic groups, the category 'other race and ethnicity' included American Indian or Alaska Native, Native Hawaiian or Other Pacific Islander, and individuals identifying with multiple races and ethnicities.

Missingness: We employed median imputation to handle missing values. Across variables, electronic health information was missing in 0-10% of cases. Insurance data was absent for 28% of individuals. We considered, but ultimately omitted, variables related to sexual orientation and gender identity due to high missingness and skew toward children receiving outpatient care within the health system.

Hyperparameter Optimization and Tuning: Each classifier's hyperparameters were tuned through grid search with nested cross-validation. The following hyperparameters and values were considered: Maximum depth (3, 5, 10, none), maximum features (sqrt, log2, and none), minimum leaf samples (1, 2, 3), minimum split (2, 5, 10), and n estimators (100, 200, 300). Tuning occurred exclusively on training

© 2024 Edgcomb JB et al. *JAMA Network Open.*

data; no test data was used to tune the classifier. Class weights were adjusted to be inversely proportional to the number of samples in each class (balanced).

Comparator classifier: We selected random forest classifiers due to robustness to overfitting, missingness, and efficiency. A lasso-penalized logistic regression classifier was trained and tested using the same method to demonstrate comparable performance across classifier types.

Toolboxes: sklearn.ensemble.RandomForestClassifier, sklearn.multiclass, sklearn.model_selection.GridSearchCV, and sklearn.metrics.

© 2024 Edgcomb JB et al. *JAMA Network Open.*

eTable 1. Mental Health-related Chief Concerns in Structured Data Fields

Agitation
Altered Mental Status
Psychiatric Evaluation
Aggressive Behavior
Alcohol Intoxication
Depression
Agitation
Manic Behavior
Hallucinations
Homicidal
Panic Attack
Behavior Problem
Anxiety
Delusional
Eating Disorder
Support For Psychological Distress
Suicidal
Suicide Attempt
Poisoning
Drug Overdose
Ingestion

© 2024 Edgcomb JB et al. *JAMA Network Open.*

eTable 2. Full Sample Characteristics by Age Group

	Total		Children (6-12y)		Adolescents (13-17y)	
	n	%	n	%	n	%
**Gold-standard**	2702		898	33.2	1804	66.8
Any SITB	1286	47.6	427	47.6	859	47.6
Suicide Attempt	225	8.3	58	6.5	167	9.3
Preparatory Acts	189	7.0	71	7.9	118	6.5
Suicidal Ideation	749	27.7	233	25.9	516	28.6
NSSI	543	20.1	181	20.2	362	20.1
Other Reason for Visit	1397	51.7	463	51.6	934	51.8
Not Enough Information	19	0.7	8	0.9	11	0.6
**Sex**						
Female	1384	51.2	359	40.0	1025	56.8
**Race and Ethnicity**						
Not Hispanic or Latino						
American Indian or Alaska Native	6	0.2	0	0.0	6	0.3
Asian	131	4.8	38	4.2	93	5.2
Black or African American	266	9.8	107	11.9	159	8.8
Multiple Races	68	2.5	24	2.7	44	2.4
Native Hawaiian or Other Pacific Islander	2	0.1	0	0.0	2	0.1
White	1319	48.8	403	44.9	916	50.8
Other	157	6.1	62	6.9	95	5.2
Hispanic or Latino	719	26.6	253	28.2	466	25.8
Unknown	34	1.3	11	1.2	23	1.3
**Site**						
Academic medical center	2021	74.8	728	81.1	1293	71.7
Community hospital	681	25.2	170	18.9	511	28.3
**Disposition**						
Discharged without hospitalization	1558	57.7	517	57.6	1041	57.7
General medical hospitalization	334	12.4	121	13.5	213	11.8
Psychiatric hospitalization	746	27.6	234	26.1	512	28.4
Within health system	566	20.9	195	21.7	371	20.6
Transferred outside health system	180	6.7	39	4.3	141	7.8
**Chief complaint**						
Psychiatric (including suicide-related)	1516	56.1	534	59.5	982	54.4
Suicide-related	707	26.2	165	18.4	542	30.0
**Legal status**						
Involuntary mental health detainment	636	23.5	206	22.9	430	23.8
Detainment Continued by MH Provider	271	10.0	55	6.1	216	12.0
Detainment Discontinued	287	10.6	82	9.1	205	11.4
Voluntary	2066	76.5	692	77.1	1374	76.2
**ED Diagnostic Code Category (CAMHD-CS)**						
ADHD	578	21.4	272	30.3	306	17.0
Anxiety disorders	768	28.4	207	23.1	561	31.1

© 2024 Edgcomb JB et al. *JAMA Network Open.*

Autism spectrum disorder	321	11.9	155	17.3	166	9.2
Bipolar and related disorders	106	3.9	38	4.2	68	3.8
Communication disorders	25	0.9	18	2.0	7	0.4
Depressive disorders	943	34.9	196	21.8	747	41.4
Developmental delay or unspecified neurodevelopmental disorder	73	2.7	44	4.9	29	1.6
Disruptive, impulse control and conduct disorders	202	7.5	134	14.9	68	3.8
Feeding and eating disorders	60	2.2	9	1.0	51	2.8
Intellectual disability	48	1.8	20	2.2	28	1.6
Mental health symptom	369	13.7	193	21.5	176	9.8
Miscellaneous	141	5.2	59	6.6	82	4.5
Motor disorders	30	1.1	20	2.2	10	0.6
Neurocognitive disorders	38	1.4	12	1.3	26	1.4
Obsessive-compulsive and related disorders	121	4.5	39	4.3	82	4.5
Personality disorders	19	0.7	4	0.4	15	0.8
Schizophrenia spectrum and other psychotic disorders	90	3.3	28	3.1	62	3.4
Sexuality and gender identity disorders	24	0.9	2	0.2	22	1.2
Specific learning disorders	18	0.7	9	1.0	9	0.5
Substance related and addictive disorders	237	8.8	8	0.9	229	12.7
Suicide or self-injury	950	35.2	266	29.6	684	37.9
Trauma and stressor-related disorders	167	6.2	65	7.2	102	5.7
Psychiatric Comorbidity (≥ 2 CAMHD-CS diagnostic groups)	1657	61.3	598	66.6	1059	58.7
**Acute Care Use**						
Prior ED use (≥ 1 visit)						
Past 90 days	601	22.2	194	21.6	407	22.6
Past 180 days	967	35.8	309	34.4	658	36.5
Past 365 days	1577	58.4	457	50.9	1120	62.1
Prior Medical Hospitalization (≥1 visit)						
Past 90 days	97	3.6	31	3.5	66	3.7
Past 180 days	166	6.1	50	5.6	116	6.4
Past 365 days	279	10.3	80	8.9	199	11.0
Prior Psychiatric Hospitalization (≥ 1 visit)						
Past 90 days	99	3.7	36	4.0	63	3.5
Past 180 days	173	6.4	58	6.5	115	6.4
Past 365 days	260	9.6	79	8.8	181	10.0
**Medications received during ED visit**						
Antidepressant	549	20.3	143	15.9	406	22.5
Antiepileptic	117	4.3	46	5.1	71	3.9
Antihistamine	100	3.7	29	3.2	71	3.9
Antipsychotic	439	16.2	159	17.7	280	15.5
Anxiolytic	239	8.8	47	5.2	192	10.6
Hypnotic or sedative	43	1.6	16	1.8	27	1.5
Lithium	42	1.6	11	1.2	31	1.7
Psychostimulant	233	8.6	126	14.0	107	5.9
Injectable medication	84	3.1	14	1.6	70	3.9

© 2024 Edgcomb JB et al. *JAMA Network Open.*

Urine drug screen results (Positive)						
Amphetamine	43	1.6	7	0.8	36	2.0
Benzodiazepines	34	1.3	0	0.0	34	1.9
Cannabis	122	4.5	1	0.1	121	6.7
Cocaine	4	0.1	0	0.0	4	0.2
Opiates	4	0.1	0	0.0	4	0.2
Ethanol	24	0.9	0	0.0	24	1.3
Insurance Status						
Private	1032	38.2	225	25.1	807	44.7
Public	614	22.7	199	22.2	415	23.0
Other or missing	767	28.4	201	22.4	566	31.4

	**Median (IQR)**	**Median (IQR)**	**Median (IQR)**
**Age, y**	14 (12-16)	11 (10-12)	16 (14-17)
**Social Vulnerability Index, Total**	0.378 (0.187-0.645)	0.422 (0.199-0.710)	0.366 (0.185-0.621)
Socioeconomic Status	0.294 (0.137-0.569)	0.301 (0.146-0.660)	0.290 (0.221-0.673)
Household Composition & Disability	0.232 (0.110-0.425)	0.275 (0.122-0.470)	0.221 (0.098-0.420)
Minority Status & Language	0.684 (0.550-0.847)	0.723 (0.559-0.869)	0.672 (0.546-0.827)
Housing Type & Transportation	0.507 (0.244-0.749)	0.499 (0.241-0.767)	0.508 (0.246-0.748)
**Area Deprivation Index**			
State Ranking	2 (1-5)	3 (1-5)	2 (1-4)
National Ranking	5 (2-12)	6 (2-14)	4 (2-11)

© 2024 Edgcomb JB et al. *JAMA Network Open.*

eTable 3. Random Forest Classifier Performance with Table of Counts and Fit Metrics, by Age Group and Sex, Race, and Ethnicity

	Case Surveillance (CS) Classifier									Augmented Case Surveillance (aCS) Classifier									
**Train on all**	**TP**	**FP**	**FN**	**TN**	**Total**	**Sensitivity (95% CI)**	**Specificity (95% CI)**	**Accuracy (95% CI)**	**AUC-ROC (95% CI)**	**TP**	**FP**	**FN**	**TN**	**Total**	**Sensitivity (95% CI)**	**Specificity (95% CI)**	**Accuracy (95% CI)**	**AUC-ROC (95% CI)**	**P-value[a]**
All (6-17yo)	1024	20	262	1396	2702	0.796 (0.771-0.821)	0.985 (0.979-0.992)	0.895 (0.884-0.907)	0.894 (0.882-0.905)	1183	149	103	1267	2702	0.920 (0.905-0.935)	0.894 (0.877-0.911)	0.906 (0.895-0.918)	0.975 (0.968-0.980)	< 0.001
Children	286	4	141	467	898	0.67 (0.623-0.714)	0.992 (0.978-0.998)	0.839 (0.813-0.862)	0.841 (0.815-0.867)	375	68	52	403	898	0.878 (0.843-0.908)	0.856 (0.821-0.886)	0.866 (0.842-0.888)	0.956 (0.942-0.97)	< 0.001
Adolescents	738	16	121	929	1804	0.859 (0.834-0.882)	0.983 (0.973-0.99)	0.924 (0.911-0.936)	0.925 (0.912-0.938)	808	81	51	864	1804	0.941 (0.923-0.955)	0.914 (0.895-0.931)	0.927 (0.914-0.938)	0.981 (0.974-0.988)	< 0.001
Female	631	6	107	640	1384	0.855 (0.828-0.88)	0.991 (0.98-0.997)	0.918 (0.903-0.932)	0.923 (0.909-0.937)	694	49	44	597	1384	0.94 (0.921-0.956)	0.924 (0.901-0.943)	0.933 (0.918-0.945)	0.985 (0.979-0.991)	< 0.001
Male	393	14	155	756	1318	0.717 (0.677-0.755)	0.982 (0.97-0.99)	0.872 (0.853-0.889)	0.869 (0.848-0.89)	489	100	59	670	1318	0.892 (0.863-0.917)	0.87 (0.844-0.893)	0.879 (0.861-0.896)	0.961 (0.949-0.973)	< 0.001
Asian	53	2	11	65	131	0.828 (0.713-0.911)	0.97 (0.896-0.996)	0.901 (0.836-0.946)	0.895 (0.838-0.952)	58	9	6	58	131	0.906 (0.807-0.965)	0.866 (0.76-0.937)	0.885 (0.818-0.934)	0.970 (0.940-1.000)	0.005
White	560	7	134	618	1319	0.807 (0.776-0.836)	0.989 (0.977-0.995)	0.893 (0.875-0.909)	0.901 (0.884-0.918)	647	87	47	538	1319	0.932 (0.911-0.95)	0.861 (0.831-0.887)	0.898 (0.881-0.914)	0.973 (0.964-0.982)	< 0.001
Black	94	2	34	136	266	0.734 (0.649-0.809)	0.986 (0.949-0.998)	0.865 (0.818-0.903)	0.859 (0.813-0.905)	117	17	11	121	266	0.914 (0.851-0.956)	0.877 (0.81-0.927)	0.895 (0.851-0.929)	0.965 (0.942-0.988)	< 0.001
Other Race or Ethnicity	81	2	15	135	233	0.844 (0.755-0.91)	0.985 (0.948-0.998)	0.927 (0.886-0.957)	0.895 (0.85-0.94)	92	11	4	126	233	0.958 (0.897-0.989)	0.92 (0.861-0.959)	0.936 (0.896-0.964)	0.986 (0.969-1.003)	0.003
Hispanic or Latino	223	7	65	424	719	0.774 (0.722-0.821)	0.984 (0.967-0.993)	0.9 (0.876-0.921)	0.861 (0.831-0.891)	253	23	35	408	719	0.878 (0.835-0.914)	0.947 (0.921-0.966)	0.919 (0.897-0.938)	0.98 (0.968-0.992)	< 0.001
Children (6-12yo)																			
Female	147	0	45	167	359	0.766 (0.699-0.824)	1 (0.978-1)	0.875 (0.836-0.907)	0.880 (0.845-0.915)	174	17	18	150	359	0.906 (0.856-0.943)	0.898 (0.842-0.94)	0.903 (0.867-0.931)	0.979 (0.964-0.994)	< 0.001
Male	139	4	96	300	539	0.591 (0.526-0.655)	0.987 (0.967-0.996)	0.814 (0.779-0.846)	0.814 (0.776-0.852)	201	51	34	253	539	0.855 (0.804-0.898)	0.832 (0.785-0.872)	0.842 (0.809-0.872)	0.939 (0.917-0.961)	< 0.001
Asian	13	2	6	17	38	0.684 (0.434-0.874)	0.895 (0.669-0.987)	0.789 (0.627-0.904)	0.824 (0.689-0.959)	15	4	4	15	38	0.789 (0.544-0.939)	0.789 (0.544-0.939)	0.789 (0.627-0.904)	0.911 (0.813-1.009)	0.153

© 2024 Edgcomb JB et al. *JAMA Network Open.*

White	149	1	70	183	403	0.68 (0.614-0.742)	0.995 (0.97-1)	0.824 (0.783-0.86)	0.815 (0.774-0.856)	194	38	25	146	403	0.886 (0.836-0.925)	0.793 (0.728-0.85)	0.844 (0.804-0.878)	0.943 (0.920-0.966)	< 0.001
Black	33	0	22	52	107	0.6 (0.459-0.73)	1 (0.932-1)	0.794 (0.705-0.866)	0.791 (0.706-0.876)	50	10	5	42	107	0.909 (0.8-0.97)	0.808 (0.675-0.904)	0.86 (0.779-0.919)	0.941 (0.895-0.987)	< 0.001
Other Race or Ethnicity	22	0	9	55	86	0.71 (0.52-0.858)	1 (0.935-1)	0.895 (0.811-0.951)	0.799 (0.695-0.903)	29	4	2	51	86	0.935 (0.786-0.992)	0.927 (0.824-0.98)	0.93 (0.854-0.974)	0.982 (0.948-1.016)	< 0.001
Hispanic or Latino	66	1	32	154	253	0.673 (0.571-0.765)	0.994 (0.965-1)	0.870 (0.822-0.908)	0.812 (0.755-0.869)	82	11	16	144	253	0.837 (0.748-0.904)	0.929 (0.877-0.964)	0.893 (0.849-0.928)	0.975 (0.953-0.997)	< 0.001
Adolescents (13-17yo)																			
Female	484	6	62	473	1025	0.886 (0.857-0.912)	0.987 (0.973-0.995)	0.934 (0.917-0.948)	0.939 (0.924-0.954)	520	32	26	447	1025	0.952 (0.931-0.969)	0.933 (0.907-0.954)	0.943 (0.927-0.957)	0.987 (0.98-0.994)	< 0.001
Male	254	10	59	456	779	0.812 (0.764-0.853)	0.979 (0.961-0.99)	0.911 (0.889-0.93)	0.904 (0.88-0.928)	288	49	25	417	779	0.92 (0.884-0.948)	0.895 (0.863-0.921)	0.905 (0.882-0.925)	0.972 (0.959-0.985)	< 0.001
Asian	40	0	5	48	93	0.889 (0.759-0.963)	1 (0.926-1)	0.946 (0.879-0.982)	0.94 (0.889-0.991)	43	5	2	43	93	0.956 (0.849-0.995)	0.896 (0.773-0.965)	0.925 (0.851-0.969)	0.987 (0.963-1.011)	0.078
White	411	6	64	435	916	0.865 (0.831-0.895)	0.986 (0.971-0.995)	0.924 (0.904-0.94)	0.927 (0.91-0.944)	453	49	22	392	916	0.954 (0.931-0.971)	0.889 (0.856-0.917)	0.922 (0.903-0.939)	0.981 (0.972-0.99)	< 0.001
Black	61	2	12	84	159	0.836 (0.73-0.912)	0.977 (0.919-0.997)	0.912 (0.857-0.951)	0.917 (0.87-0.964)	67	7	6	79	159	0.918 (0.83-0.969)	0.919 (0.839-0.967)	0.918 (0.864-0.956)	0.977 (0.952-1.002)	0.008
Other Race or Ethnicity	59	2	6	80	147	0.908 (0.81-0.965)	0.976 (0.915-0.997)	0.946 (0.896-0.976)	0.942 (0.9-0.984)	63	7	2	75	147	0.969 (0.893-0.996)	0.915 (0.832-0.965)	0.939 (0.887-0.972)	0.988 (0.969-1.007)	0.031
Hispanic or Latino	157	6	33	270	466	0.826 (0.765-0.877)	0.978 (0.953-0.992)	0.916 (0.887-0.94)	0.896 (0.864-0.928)	171	12	19	264	466	0.9 (0.848-0.939)	0.957 (0.925-0.977)	0.933 (0.907-0.954)	0.982 (0.969-0.995)	< 0.001

**Train by age group**	**TP**	**FP**	**FN**	**TN**	**Total**	**Sensitivity (95% CI)**	**Specificity (95% CI)**	**Accuracy (95% CI)**	**AUC-ROC (95% CI)**	**TP**	**FP**	**FN**	**TN**	**Total**	**Sensitivity (95% CI)**	**Specificity (95% CI)**	**Accuracy (95% CI)**	**AUC-ROC (95% CI)**	
Children (6-12yo)	286	4	141	467	898	0.670 (0.615-0.724)	0.992 (0.983-0.999)	0.838 (0.814-0.862)	0.853 (0.830-0.876)	369	59	58	412	898	0.866 (0.832-0.901)	0.877 (0.845-0.908)	0.870 (0.848-0.891)	0.957 (0.944-0.970)	< 0.001
Adolescents (13-17yo)	733	16	126	929	1804	0.855 (0.829-0.880)	0.982 (0.974-0.991)	0.921 (0.909-0.934)	0.926 (0.9213-0.938)	803	75	56	870	1804	0.936 (0.919-0.952)	0.921 (0.903-0.939)	0.927 (0.915-0.939)	0.980 (0.973-0.986)	< 0.001

© 2024 Edgcomb JB et al. *JAMA Network Open.*

Lasso Logistic Regression Comparator	TP	FP	FN	TN	Total	Sensitivity (95% CI)	Specificity (95% CI)	Accuracy (95% CI)	AUC-ROC	TP	FP	FN	TN	Total	Sensitivity (95% CI)	Specificity (95% CI)	Accuracy (95% CI)	AUC-ROC	
All (6-17yo)	1024	20	262	1396	2702	0.796 (0.773-0.818)	0.985 (0.978-0.991)	0.896 (0.883-0.907)	0.906 (0.894-0.910)	1139	108	147	1308	2702	0.886 (0.867-0.902)	0.923 (0.909-0.937)	0.906 (0.894-0.916)	0.968 (0.961-0.975)	< 0.001
Children (6-12yo)	286	4	141	467	898	0.670 (0.615-0.724)	0.992 (0.983-0.999)	0.838 (0.814-0.862)	0.853 (0.827-0.879)	358	53	69	418	898	0.838 (0.808-0.872)	0.887 (0.855-0.914)	0.864 (0.840-0.885)	0.945 (0.931-0.959)	< 0.001
Adolescents (13-17yo)	738	16	121	929	1804	0.859 (0.834-0.882)	0.983 (0.972-0.990)	0.924 (0.911-0.936)	0.922 (0.909-0.935)	781	55	78	890	1804	0.909 (0.888-0.927)	0.941 (0.925-0.956)	0.926 (0.913-0.938)	0.976 (0.969-0.983)	< 0.001

a. DeLong test to detect statistically significant difference in AUC-ROC of CS and aCS Classifiers, b. Other Race and Ethnicity includes American Indian or Alaska Native, Native Hawaiian or Other Pacific Islander, and multiple races and ethnicities; excludes unknown

© 2024 Edgcomb JB et al. *JAMA Network Open.*

eTable 4. DeLong test comparing augmented case surveillance performance by age group of training set

		P-value	Z
Train on 6-17 / Test on 6-12	Train on 6-12 / Test on 6-12	0.9901819	-0.0123072
Train on 6-17 / Test on 13-17	Train on 13-17 / Test on 13-17	0.47051107	-0.7217266

© 2024 Edgcomb JB et al. *JAMA Network Open.*

eTable 5. Comparison of Performance for Detection of Self-injurious Thoughts and Behaviors with Table of Counts and Fit Metrics, by Type and by Age Group

	Case Surveillance (CS) Classifier								Augmented Case Surveillance (aCS) Classifier								P-value
	TP	FP	FN	TN	Sensitivity (95% CI)	Specificity (95% CI)	Accuracy (95% CI)	AUC-ROC (95% CI)	TP	FP	FN	TN	Sensitivity (95% CI)	Specificity (95% CI)	Accuracy (95% CI)	AUC-ROC (95% CI)	
All (6-17yo)																	
Suicidality																	
Attempt or Act	200	333	214	1955	0.482 (0.413-0.551)	0.854 (0.839-0.870)	0.797 (0.782-0.812)	0.792 (0.776-0.807)	178	207	236	2081	0.436 (0.363-0.508)	0.912 (0.901-0.925)	0.839 (0.825-0.853)	0.859 (0.846-0.873)	< 0.001
Ideation	308	205	441	1748	0.415 (0.360-0.470)	0.895 (0.881-0.910)	0.761 (0.745-0.777)	0.733 (0.716-0.750)	418	236	331	1717	0.562 (0.515-0.609)	0.882 (0.867-0.898)	0.793 (0.779-0.809)	0.868 (0.855-0.881)	< 0.001
None	1478	178	61	985	0.960 (0.950-0.970)	0.847 (0.824-0.869)	0.911 (0.901-0.922)	0.919 (0.910-0.930)	1470	193	69	970	0.963 (0.953-0.972)	0.838 (0.815-0.861)	0.909 (0.898-0.920)	0.971 (0.973-0.984)	< 0.001
Non-suicidal self-injury	250	440	293	1719	0.461 (0.400-0.533)	0.796 (0.777-0.815)	0.728 (0.712-0.745)	0.638 (0.712-0.745)	62	50	481	2109	0.114 (0.035-0.193)	0.977 (0.970-0.983)	0.803 (0.788-0.818)	0.801 (0.786-0.816)	< 0.001
Children (6-12yo)																	
Suicidality																	
Attempt or Act	57	84	72	685	0.442 (0.355-0.532)	0.891 (0.867-0.912)	0.826 (0.800-0.851)	0.741 (0.690-0.792)	52	57	77	712	0.403 (0.318-0.493)	0.926 (0.905-0.943)	0.851 (0.826-0.873)	0.851 (0.808-0.893)	< 0.001
Ideation	93	60	140	605	0.399 (0.336-0.465)	0.910 (0.885-0.930)	0.777 (0.749-0.804)	0.728 (0.687-0.768)	120	65	113	600	0.515 (0.449-0.581)	0.902 (0.877-0.924)	0.801 (0.774-0.827)	0.853 (0.821-0.885)	< 0.001
None	513	91	23	271	0.957 (0.936-0.973)	0.749 (0.701-0.792)	0.873 (0.849-0.894)	0.857 (0.833-0.880)	513	91	23	271	0.957 (0.936-0.972)	0.748 (0.701-0.792)	0.873 (0.849-0.894)	0.945 (0.930-0.959)	< 0.001
Non-suicidal self-injury	46	94	135	623	0.254 (0.192-0.324)	0.869 (0.841-0.892)	0.745 (0.715-0.773)	0.591 (0.543-0.639)	11	15	170	702	0.061 (0.0.03-0.106)	0.979 (0.965-0.988)	0.793 (0.766-0.820)	0.761 (0.718-0.804)	< 0.001
Adolescents (13-17yo)																	
Suicidality																	
Attempt or Act	142	244	143	1275	0.498 (0.439-0.558)	0.839 (0.820-0.857)	0.785 (0.766-0.804)	0.808 (0.776-0.839)	969	97	34	704	0.449 (0.390-0.509)	0.906 (0.890-0.920)	0.834 (0.816-0.851)	0.861 (0.844-0.878)	< 0.001
Ideation	217	148	299	1140	0.421 (0.378-0.464)	0.885 (0.866-0.902)	0.752 (0.732-0.772)	0.728 (0.701-0.755)	302	165	214	1123	0.585 (0.541-0.628)	0.872 (0.852-0.890)	0.790 (0.770-0.809)	0.871 (0.850-0.891)	< 0.001

© 2024 Edgcomb JB et al. *JAMA Network Open.*

None	966	87	37	714	0.963 (0.950-0.974)	0.891 (0.868-0.912)	0.931 (0.919-0.943)	0.943 (0.932-0.953)	128	143	157	1376	0.966 (0.953-0.976)	0.879 (0.854-0.901)	0.927 (0.914-0.939)	0.977 (0.964-0.989)	< 0.001
Non-suicidal self-injury	204	346	158	204	0.563 (0.511-0.615)	0.760 (0.737-0.781)	0.721 (0.699-0.741)	0.662 (0.625-0.698)	51	35	311	1407	0.141 (0.107-0.181)	0.975 (0.966-0.983)	0.808 (0.789-0.826)	0.818 (0.790-0.845)	< 0.001

© 2024 Edgcomb JB et al. *JAMA Network Open.*

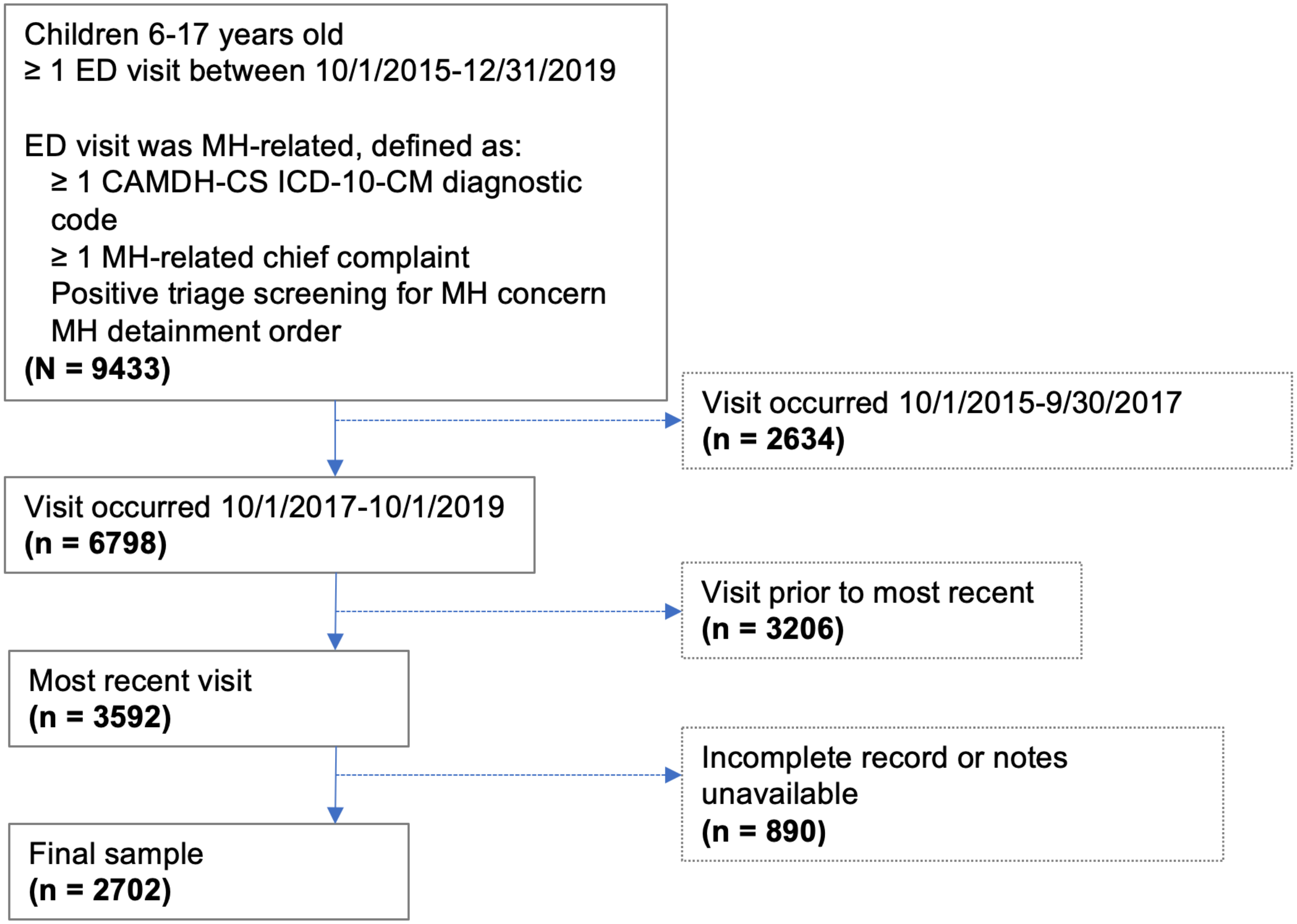

eFigure 1. Flow Diagram for Study Inclusion
ED: Emergency Department, CAMDH-CS: Child and Adolescent Mental Health Disorders Classification System, ICD-10-CM: International Classification of Diseases, Version 10, Clinical Modification, MH: Mental Health.

© 2024 Edgcomb JB et al. *JAMA Network Open.*

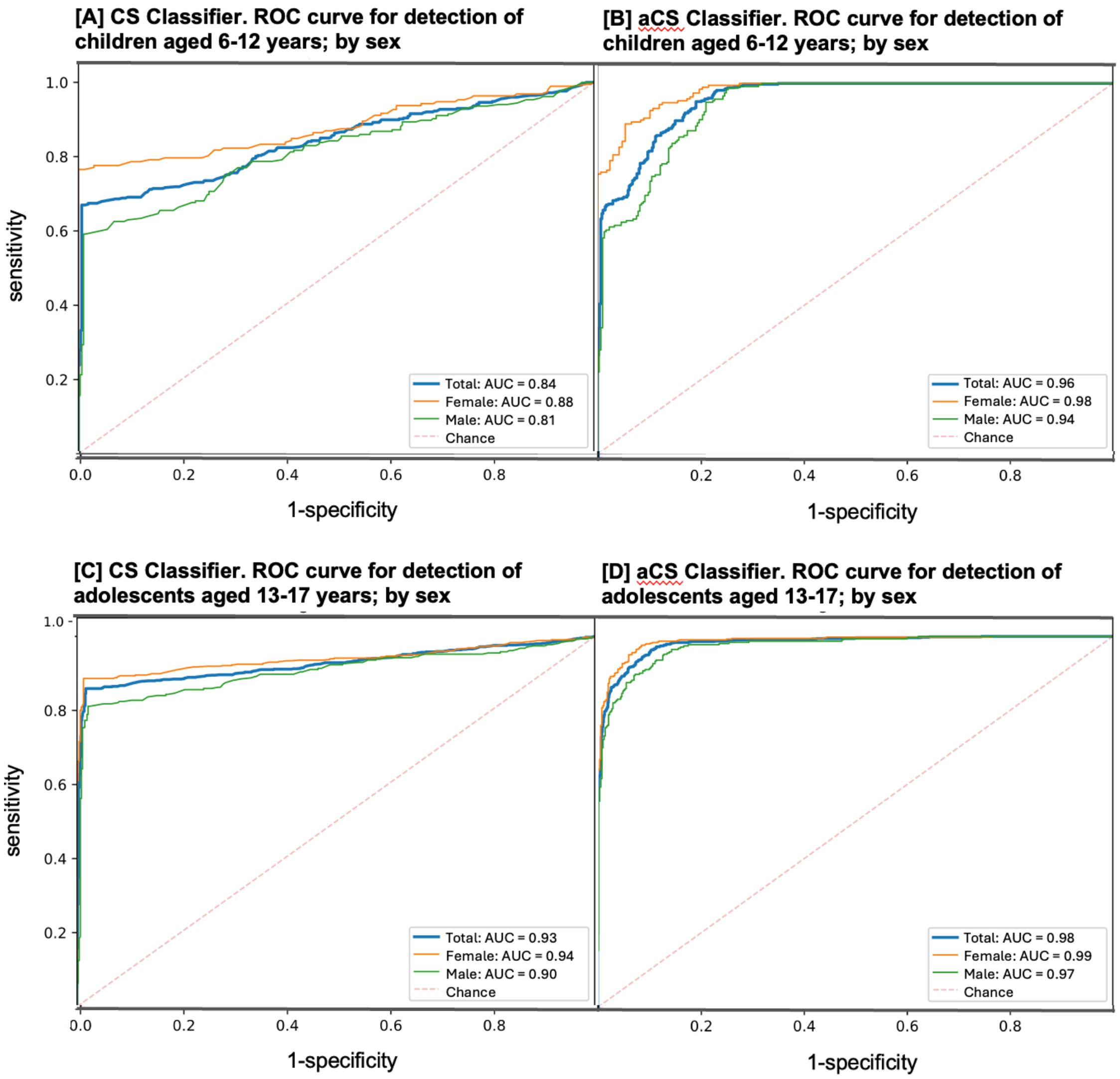

eFigure 2. Receiver Operating Characteristic (ROC) Curves by Sex.
The diagram is a composite of ROC curves for Case Surveillance (CS) and Augmented Case Surveillance (aCS) random forest classifiers for detection of self-injurious thoughts and behaviors (SITB) among youth aged 6-17, displaying performance by fold. Features of CS classifiers include Centers for Disease Control and Prevention Case Surveillance ICD-10-CM code list for non-fatal suicide attempt and self-harm, suicide-related chief complaint, and individual age, sex, race and ethnicity. Features of aCS classifiers adds structured data elements from individual electronic health records, including medications administered, laboratory testing, emergency department disposition, and mental health detainment. [A] CS Classifier and [B] aCS Classifier performance when trained on data from children aged 6-12 (N=898), and [C] CS Classifier and [D] aCS Classifier performance when trained on data from adolescents aged 13-17 (N=1208).

© 2024 Edgcomb JB et al. *JAMA Network Open.*

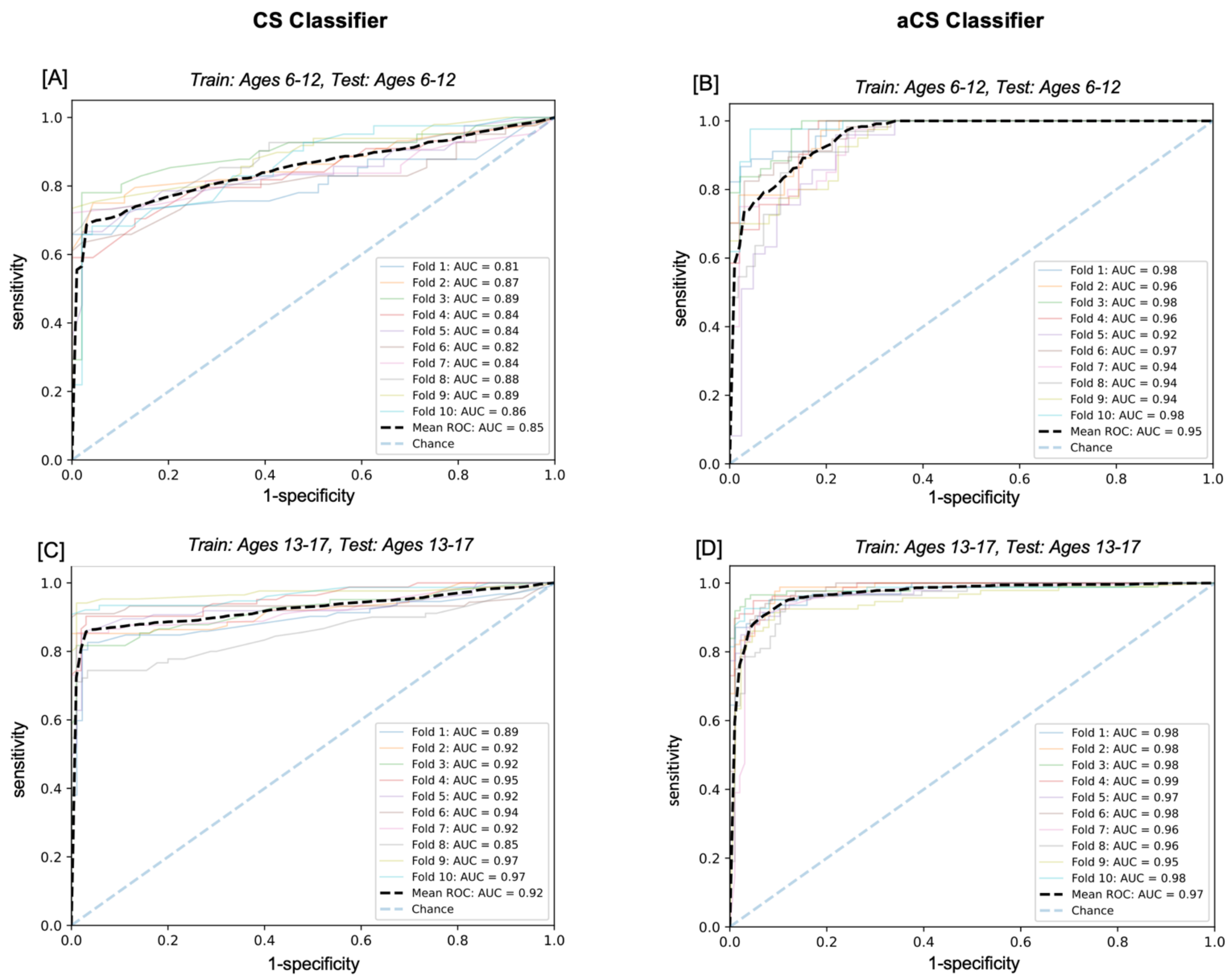

eFigure 3. Receiver Operating Characteristic (ROC) Curves by Age Group of Training Set

The diagram is a composite of ROC curves for Case Surveillance (CS) and Augmented Case Surveillance (aCS) random forest classifiers for detection of self-injurious thoughts and behaviors (SITB) among youth aged 6-17, displaying performance by fold. Features of CS classifiers include Centers for Disease Control and Prevention Case Surveillance ICD-10-CM code list for non-fatal suicide attempt and self-harm, suicide-related chief complaint, and individual age, sex, race and ethnicity. Features of aCS classifiers adds structured data elements from individual electronic health records, including medications administered, laboratory testing, emergency department disposition, and mental health detainment. [A] CS Classifier and [B] aCS Classifier performance when trained on data from children aged 6-12 (N=898), and [C] CS Classifier and [D] aCS Classifier performance when trained on data from adolescents aged 13-17 (N=1208).

© 2024 Edgcomb JB et al. *JAMA Network Open.*

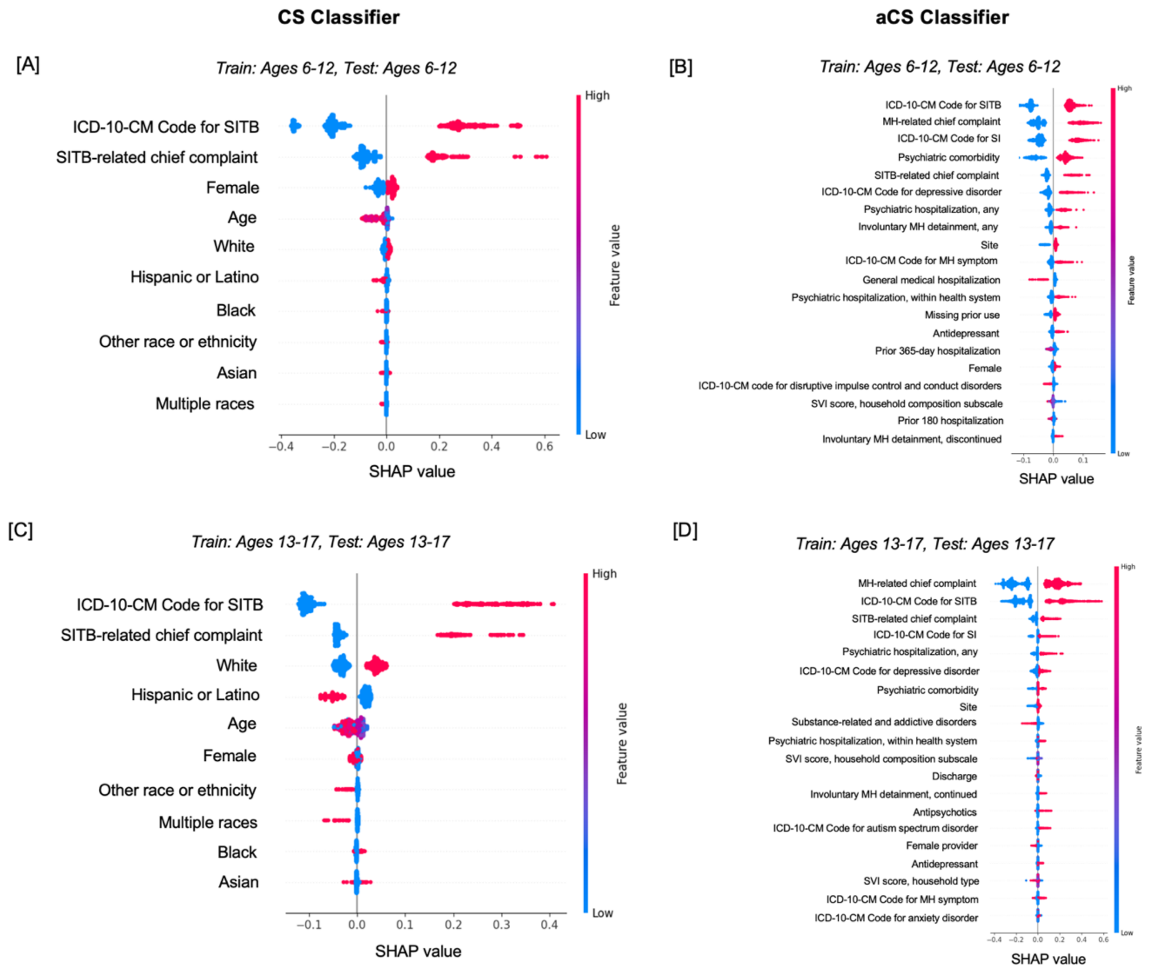

eFigure 4. SHapley Additive exPlanations (SHAP) Plots by Age Group of Training Set

The diagram is a composite of SHAP plots for Case Surveillance (CS) [left] and Augmented Case Surveillance (aCS) [right] random forest classifiers for detection of self-injurious thoughts and behaviors (SITB) among youth aged 6-17. Features of CS classifiers include Centers for Disease Control and Prevention Case Surveillance International Classification of Diseases, Version 10, Clinical Modification (ICD-10-CM) code list for non-fatal suicide attempt and self-harm, suicide-related chief complaint, and individual age, sex, race and ethnicity. Features of aCS classifiers adds structured data elements from individual electronic health records, including medications administered, laboratory testing, emergency department disposition, and mental health detainment. For each classifier, features are shown in descending order of feature importance. [A] CS Classifier and [B] aCS Classifier performance when trained on data from children aged 6-12 (N=898), and [C] CS Classifier and [D] aCS Classifier performance when trained on data from adolescents aged 13-17 (N=1208). *SI: Suicidal Ideation, MH: Mental Health, SVI: Social Vulnerability Index.*

© 2024 Edgcomb JB et al. *JAMA Network Open.*

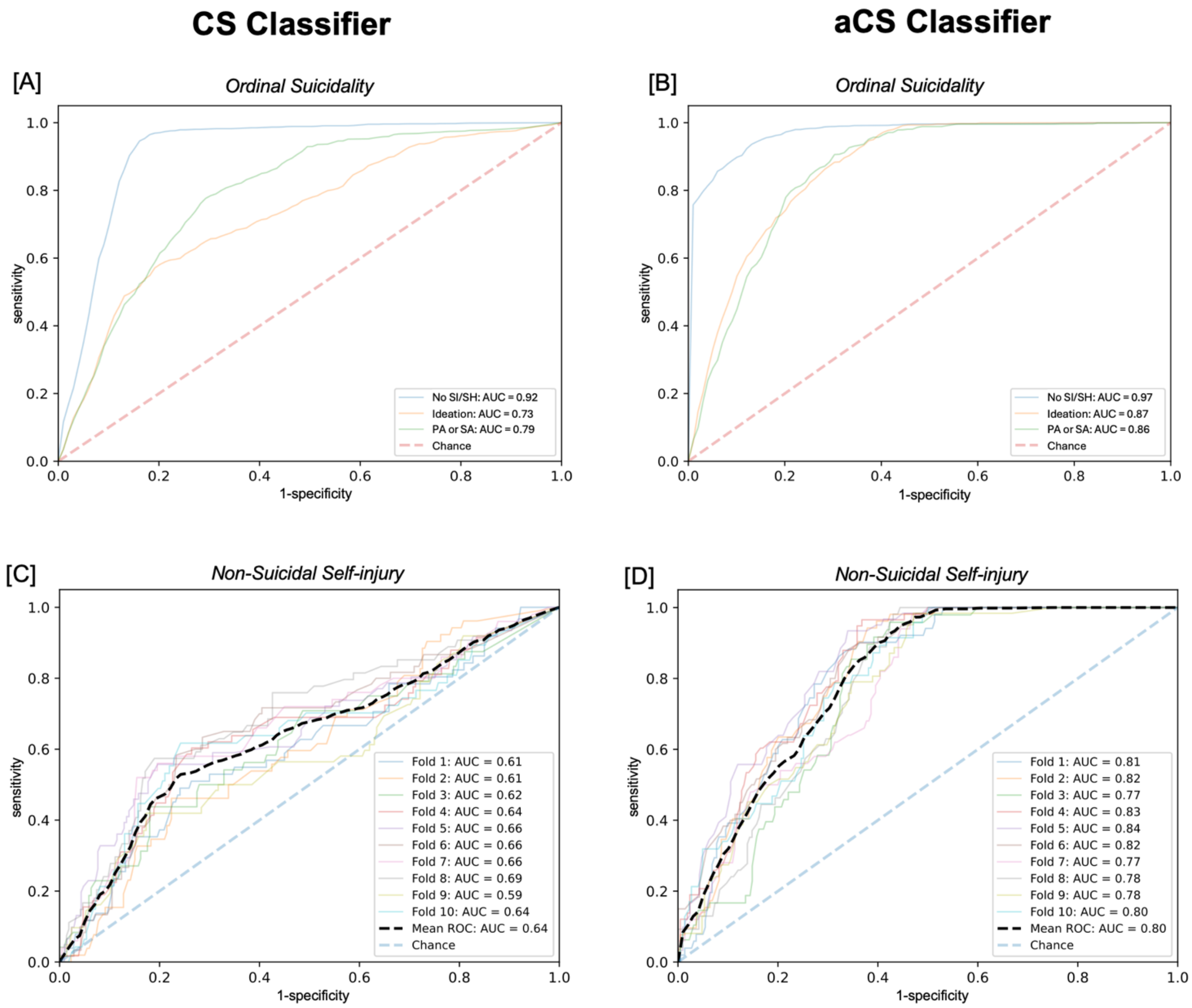

eFigure 5. Receiver Operating Characteristic (ROC) Curves for Detection of Self-injurious Thoughts and Behavior by Type

The diagram is a composite of ROC curves for Case Surveillance (CS) [left] and Augmented Case Surveillance (aCS) [right] random forest classifiers for detection of ordinal suicidality [A,B] and non-suicidal self-injury [C,D]. Ordinal suicidality classes are no suicidal ideation or self-harm (blue), suicidal ideation (orange), and preparatory acts or suicide attempt (green) and lines represent the mean ROC of each class across folds. *SI/SH: Suicidal ideation or self-harm*, *PA: preparatory act, SA: suicide attempt*.

© 2024 Edgcomb JB et al. *JAMA Network Open.*
