# Supplementary material for: Electronic Health Record Phenotyping of Pediatric Suicide-Related Emergency Department Visits
Source: JAMA Netw Open. 2024 Oct 29;7(10):e2442091. doi: 10.1001/jamanetworkopen.2024.42091 (PMC11522940; doi:10.1001/jamanetworkopen.2024.42091)
Supplement: Supplement 2. — Data Sharing Statement [file jamanetwopen-e2442091-s002.pdf]

Data Sharing Statement

Edgcomb. Electronic Health Record Phenotyping of Pediatric Suicide-Related Emergency Department Visits. *JAMA Netw Open*. Published online October 29, 2024. doi:10.1001/jamanetworkopen.2024.42091

Data
Data available: No

Additional Information
Explanation for why data not available: The use of protected health information (PHI) from verbatim clinical notes entails strict confidentiality and privacy regulations to safeguard the sensitive information of individuals, in accordance with legal and ethical standards governing healthcare data. The nature of our dataset containing PHI prohibits its release to maintain compliance with regulatory requirements and to uphold patient confidentiality.
